# Flexible ceramic nanofibrous sponges with hierarchically entangled graphene networks enable noise absorption

**DOI:** 10.1038/s41467-021-26890-9

**Published:** 2021-11-15

**Authors:** Dingding Zong, Leitao Cao, Xia Yin, Yang Si, Shichao Zhang, Jianyong Yu, Bin Ding

**Affiliations:** 1 State Key Laboratory for Modification of Chemical Fibers and Polymer Materials, College of Materials Science and Engineering, Donghua University, 201620 Shanghai, China; 2 Innovation Center for Textile Science and Technology, Donghua University, 200051 Shanghai, China

**Keywords:** Graphene, Nanoscale materials, Ceramics

## Abstract

Traffic noise pollution has posed a huge burden to the global economy, ecological environment and human health. However, most present traffic noise reduction materials suffer from a narrow absorbing band, large weight and poor temperature resistance. Here, we demonstrate a facile strategy to create flexible ceramic nanofibrous sponges (FCNSs) with hierarchically entangled graphene networks, which integrate unique hierarchical structures of opened cells, closed-cell walls and entangled networks. Under the precondition of independent of chemical crosslinking, high enhancement in buckling and compression performances of FCNSs is achieved by forming hierarchically entangled structures in all three-dimensional space. Moreover, the FCNSs show enhanced broadband noise absorption performance (noise reduction coefficient of 0.56 in 63–6300 Hz) and lightweight feature (9.3 mg cm^–3^), together with robust temperature-invariant stability from –100 to 500 °C. This strategy paves the way for the design of advanced fibrous materials for highly efficient noise absorption.

## Abstract (web summary)

Noise pollution has been a burden to the global economy, environment, and human health. Here the authors demonstrate a facile route to produce flexible ceramic nanofibrous sponges with hierarchically entangled graphene networks and its excellent noise absorption properties at elevated temperatures.

## Introduction

With the rapid development of the transportation industry, traffic noise pollution, as a hidden killer of the global economy, ecological environment, and human health, has become increasingly serious^1,2^. According to the World Health Organization, there are >466 million people worldwide who suffer from disabling hearing loss that is mostly caused by exposure to noise, which causes total annual economic losses of >$750 billion^3^. Therefore, efficient traffic noise reduction methods are of great importance for eliminating unwanted sound. Fibrous materials with the advantages of porous structure and tortuous channels could enhance friction and dissipation of sound waves, thus showing good high-frequency (usually >1000 Hz) absorption performance^4^. Previous efforts were focused on using cost-effective fibrous materials as the core components for constructing noise-absorber^4–6^. However, due to the inherent limitations of large fiber diameter (usually >5 μm) and low porosity (<60%), the fatal defects of poor absorption of low-frequency (typically <1000 Hz) noise that is easily produced by vehicles remain for the conventional microfibrous noise-absorbing materials^7–9^. To address this problem, it is necessary to increase the thickness or density of the fibrous materials, while which in turn lead to large weights (>50 mg cm^–3^) and poor absorption of high-frequency noise^10–12^. Furthermore, the vehicle’s space left for noise-absorbing materials is limited, and increasing the density will increase fuel consumption, which violates the principle of energy saving. More importantly, the temperature of vehicles (especially in engines and firewalls) would become very high after running for a while; thus, the poor temperature resistance of commonly used polymer-based noise-absorbing materials not only easily leads to decomposing and material failure but also even causes spontaneous combustion of the vehicles^13–16^. Therefore, great efforts are required to create a specialized and practicable fibrous sound-absorbing material for the efficient reduction of broadband noise.

Compared with microfibers, electrospun nanofibers show effective improvement in noise absorption performances due to their small diameter and large specific surface area^8,17,18^. However, lamellar deposition character usually induces nanofibers to assemble into densely packed two-dimensional (2D) membranes (thickness <50 μm and porosity <80%), thus greatly limiting the dissipation of acoustic energy^19–22^. Most recently, three-dimensional (3D) nanofibrous sponges, as the forefront of advanced fibrous materials, combining small fiber diameter, high porosity (>95%), and bulk structure, have shown vast perspectives in the field of traffic noise reduction^23–26^. A few polymer-based nanofibrous noise-absorbing sponges have been successfully constructed and show enhanced noise absorption properties (e.g., noise reduction coefficient (NRC) of 0.41) in contrast to 2D membranes (NRC < 0.25)^19,27–29^. Unfortunately, due to the monotonous and uncontrollable structure, totally opened cells and highly connected macropore structure, existing nanofibrous sponges still face a narrow absorbing band (>1000 Hz) and poor compression and buckling properties^19,27^. Although the extra chemical crosslinking can improve the mechanical strength of sponges to a certain extent, the randomly distributed and unstable crosslinking points greatly limit the improvement of compression and buckling properties^19,30^. Moreover, the low decomposition temperature of polymer-based sponges (usually <150 °C) easily causes structural collapse at high temperatures, and a few present ceramic nanofibrous sponges are robust heat-resistant but suffer from stubborn brittleness with little deformation. Therefore, the challenge remains for constructing lightweight and thermostable nanofibrous noise-absorbing sponges with good structural stability and broadband noise reduction.

Herein we demonstrate a robust and facile strategy to create flexible ceramic nanofibrous sponges (FCNSs) with hierarchically entangled graphene networks by combining directional freeze-drying technology and ascorbic acid reduction method. Attributing to the unique hierarchically entangled structure composed of flexible SiO_2_ nanofibers (SNFs) and reduced graphene oxide (rGO), the obtained FCNSs present integrated features of ultralow bulk density (2 mg cm^−3^), temperature-invariant superelasticity, good bendability, and desired thermal stability. Moreover, the sandwiched lamellar architectures in the thickness direction of FCNSs enable a versatile multi-interface reflection behavior, thus achieving good broadband sound absorption performances (NRC of 0.56). Besides, the green reduction process also endows the FCNSs with enhanced moisture insulation (water contact angle of 143°). We anticipate that the successful synthesis of the sandwiched FCNSs could break the bottlenecks of the narrow absorption band (>1000 Hz) faced by traditional noise absorbers, thereby providing a broader vision for developing high-efficiency noise reduction materials.

## Results

### Fabrication and hierarchically entangled structure of FCNSs

To meet the requirements of desirable noise reduction property, desired structural stability, and good thermal endurance of traffic noise-absorbing materials, we prepared FCNSs in consideration of the following four demands: (1) There should be well-interconnected opened channels parallel to the direction of acoustic waves and closed-cell walls perpendicular to the direction of sound waves in FCNSs, which not only ensure the dissipation of sound waves but also prevent the sound wave from transmission through the materials. (2) The FCNSs should possess robust mechanical performance to guarantee their long-term application stability. (3) The FCNSs must have good thermal endurance to ensure safety in the high-temperature environment; meanwhile, materials should be lightweight to reduce the energy consumption of vehicles. (4) The FCNSs should have a constantly variable assembly architecture in the thickness direction to achieve multi-interface reflection and gradually dissipate broadband sound waves. To satisfy the first three requirements, flexible SNFs with good thermal stability were chosen as the building block to assemble fibrous framework structure; meanwhile, 2D GO nanosheets with good flexibility were selected as binder and macropore blocking agents to build effective entanglement among SNFs and block the pores of the fibrous cavity wall^20,31^. The last requirement was satisfied by designing sandwiched assembly architecture to enable multiple dissipations for broadband sound waves.

**Fig. 1:**
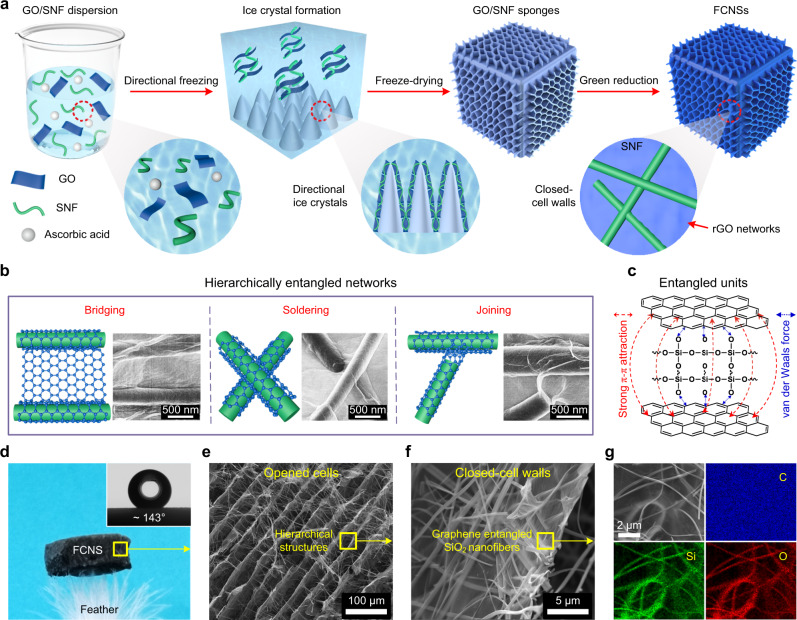
Structure design and hierarchical architectures of FCNSs. **a** Schematic illustration of the fabrication of FCNSs. **b** Schematics and corresponding SEM images of the hierarchically entangled networks. **c** The entangled unit of the crosslinking networks. **d** An optical image showing an FCNS standing on the tip of feathers. Inset: photograph of a water droplet on the FCNS. **e**, **f** The microscopic structure of FCNSs at different magnifications. **g** SEM-EDS images of a closed-cell wall with corresponding elemental mapping images.

The synthetic process of FCNSs mainly involved four components: GO, SNFs, ascorbic acid, and ultrapure water, as presented in Fig. 1a. Above all, the flexible SNFs were prepared by versatile sol–gel electrospinning technology, which possessed uniform surface morphology with an average diameter of 286 nm (Supplementary Fig. 1). Upon homogenization in water, the SNFs became well dispersed with the average fiber length of 161 μm (Supplementary Fig. 2), and the obtained SNF dispersion was mixed with GO aqueous solution by high-speed stirring. Then ascorbic acid was added as a reducing agent to the above GO/SNF dispersion for further homogenization. Subsequently, the homogenized dispersion was directional frozen in a liquid nitrogen bath and freeze-dried into GO/SNF sponges^32^. The freshly prepared GO/SNF sponges were hydrophilic due to the presence of hydrophilic groups of GO (Supplementary Fig. 3a). Finally, the obtained GO/SNF sponges were heated at 90 °C for 6 h to achieve the FCNSs with good hydrophobicity^33^ (Fig. 1d inset and Supplementary Fig. 3b). Evidence of the reduction of GO/SNF sponges was obtained by combining the Fourier transform infrared spectra (FTIR) analysis, Raman spectra analysis, and X-ray diffraction spectrum (XRD) analysis. As shown in Supplementary Fig. 4a, the absorption peaks around 3300 cm^−1^ in the infrared spectrum of GO and GO/SNF sponges were according to the hydrophilic groups in GO; no evidence for the groups was found in FCNSs, implying the successful reduction of GO/SNF sponges^34^. The relevant Raman spectra are shown in Supplementary Fig. 4b, the peaks centered at 1345 cm^–1^ and 1583 cm^–1^ were corresponding to the *D* band and *G* band, which were associated with the disordered structure and the *sp*^2^-hybridized C-C bonds, respectively. The intensity ratios of *D* and *G* peaks (*I*_*D*_/*I*_*G*_) of FCNSs were increased from 0.89 to 1.26 compared with GO/SNF sponges, indicating the formation of numerous but smaller graphitic domains after the reduction^35^. Additionally, the XRD results shown in Supplementary Fig. 4c illustrated the invisibility of the GO characteristic peak at about 10.36° in the FCNSs, further suggesting that the GO was successfully reduced to rGO^36^.

The basic entangled structure of the FCNSs consisted of one-dimensional (1D) flexible SNFs wrapped by 2D rGO networks (the enlarged detail of FCNSs in Fig. 1a). The unique entangled structure was comprised of multiform physical crosslinking networks, mainly including bridging, soldering, and joining (Fig. 1b). These crosslinking networks were attributed to strong π–π attraction among the rGO nanosheets and van der Waals force formed between the rGO nanosheets and SNFs (Fig. 1c), playing a key role in the construction of FCNSs^37,38^. Notably, we can easily adjust the volume density of FCNSs by adjusting the content of precursor GO/SNF dispersion. Typically, the FCNSs with a density of 5, 8, 12, and 18 mg cm^−3^ were named FCNS-5, FCNS-8, FCNS-12, and FCNS-18, respectively. To guarantee the good formability and desired structural stability of the sponges, the FCNSs with different mass ratios of SNF/GO (10/0, 10/1, 10/4, 10/7, and 10/10) were prepared and denoted as FCNS0, FCNS10, FCNS40, FCNS70, and FCNS100, respectively. Except for special notes, all structure and performance characterizations were performed using FCNS70 with a density of 8 mg cm^−3^ (see details in Supplementary Discussions).

Different from previous methods for preparing noise reduction materials^13,27,39,40^, our fabrication process combined the simple preparation of the SNFs with the flexibility of the directional freeze-drying technique, which enabled facile preparation and good structural tunability of FCNSs (see details in Supplementary Table 1 and Supplementary Discussions). Figure 1d indicated that a piece of FCNS weighing 6 mg (*ρ* = 2 mg cm^−3^) can easily stand on the tip of a feather, confirming its lightweight property. In sharp comparison with the beaded chain structure of common ceramic sound-absorbing aerogel, our directional freeze-drying process allowed the GO and SNFs to co-assemble into unique hierarchically entangled structures. As shown in the scanning electron microscopic (SEM) images of Fig. 1b, e–f and Supplementary Fig. 5, the hierarchical structures of FCNSs consisted of the opened cells (size of 50–100 μm), closed-cell walls (thickness of 0.5–2 μm), and entangled networks. The stable entangled structures were also confirmed by the energy-dispersive spectroscopy (EDS) mapping (Fig. 1g); the Si and O elements were completely covered with the C element, indicating that rGO was uniformly coated on the surface of the SNFs. The formation mechanism of the hierarchically entangled structures can be attributed to the phase transformation of the solvent and the co-assembly of SNFs and GO in the directional freeze-drying process^41,42^ (Fig. 1a and Supplementary Fig. 6). The solvent water solidified into ice by liquid nitrogen and formed directional ice crystals during the bottom–up freezing process (Supplementary Discussions). As the ice crystals grew, the GO nanosheets and ceramic nanofibers in the dispersion gathered along the tip of the ice crystals and were squeezed between the two icicles. Simultaneously, the GO nanosheets were gradually assembled around fibers under the extrusion of ice crystal and strong π–π interaction, which were uniformly entangled on the surface of ceramic nanofibers and firmly connected adjacent fibers. After freeze-drying, the ice crystals directly sublimated, and the aggregated GO sheets were assembled into hierarchically entangled GO networks, while ceramic nanofibers were wrapped in the networks to form 3D entangled structures. The lamellar macropores in the FCNSs were formed by the sublimation of ice crystals during the following freezing-drying process, while ceramic nanofibers were entangled by GO networks into closed-cell walls (see details in Supplementary Discussions).

### Characterization of mechanical performances of FCNSs

**Fig. 2:**
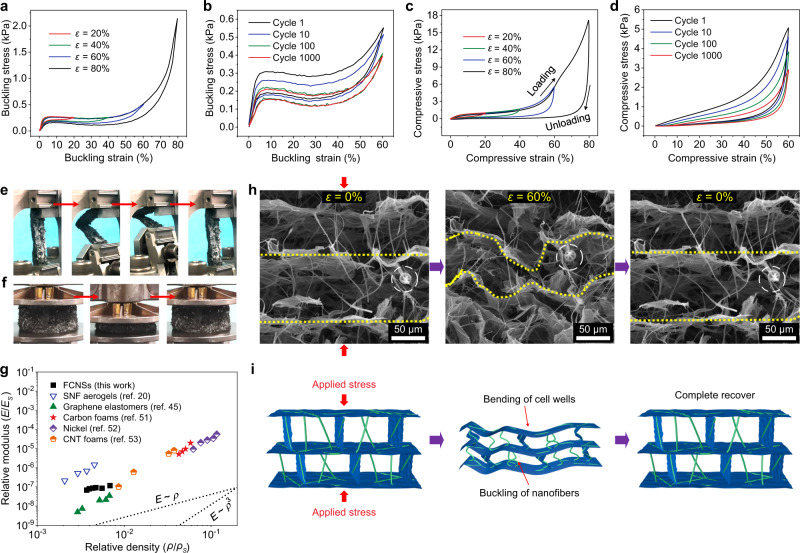
Mechanical properties of the FCNSs. **a** *σ*–*ε* curves of FCNS70 during buckling-recovery cycles with increasing *ε* amplitude. **b** A 1000-cycle bending fatigue test with *ε* = 60%. **c** Compressive *σ* versus *ε* curves during loading–unloading cycles with increasing *ε* amplitude. **d** A 1000-cycle fatigue test with compressive *ε* of 60%. Photographs of FCNS70 under **e** a buckling-recovery cycle and **f** a compressing and releasing cycle. **g** The relative Young’s modulus of selected cellular materials with low densities. **h** In situ SEM observations and **i** corresponding structure diagram of the FCNS70 under compression and release, focusing on a small piece.

The mechanical properties of the FCNSs were closely related to the content of SNFs and GO, and we discovered that the FCNS70 (SNF/GO of 10/7) could possess desired structural stability (see details in Supplementary Fig. 7a and Supplementary Discussions). In stark contrast to the poor bending properties of existing ceramic fibrous sponges^20^, the unique entangled structure endowed FCNS70 with good buckling performance, which can withstand large buckling deformation (80%) without breaking (Fig. 2e and Supplementary Movie 1). The buckling stress–strain (*σ*–*ε*) curves (Fig. 2a) of FCNS70 under different strains presented the complete closed loop, indicating the good buckling-recovery ability of FCNSs. Moreover, the maximum buckling stress under 80% strain was 2.14 kPa, higher than other ceramic nanofibrous sponges at the same densities^43^. Furthermore, the bendable FCNS70 also presented good cyclic buckling performance. As shown in Fig. 2b, no significant decrease in max stress was discovered for the FCNS70 after 1000 cyclic buckling, which can retain over 70% of the initial maximum stress (Supplementary Fig. 8a), highlighting the good buckling resistance. To our surprise, compared with the hard and fragile features of common ceramic sound-absorbing aerogels, the FCNS70 presented desirable compressive performances, which can withstand large strain without breaking (Fig. 2f and Supplementary Movie 2). Figure 2c showed the compressive *σ*–*ε* curves of FCNS70 under different strains, which exhibited typical three-stage deformation usually found in honeycomb-like materials^44^: the linear elastic deformation zone of *ε* < 6%, the platform zone of 6% < *ε* < 55% and the densification zone of *ε* > 55%, which were mainly caused by the elastic bending, elastic buckling, and densification of the cavity walls, respectively^45^. When the *ε* reached 80%, the maximum *σ* of the FCNS70 could reach 17.2 kPa, which was higher than the same type of sponges^20,41,42^, indicating that the FCNS70 possessed good impact resistance. Moreover, the FCNSs also showed desired compression fatigue resistance, as shown in Fig. 2d, and the plastic deformation of the FCNS70 was only 4.3% after 1000 compressions under a large strain (60%). Besides, Young’s modulus, energy loss coefficient, and maximum stress of the FCNS70 could remain >60% after 1000 cycles (Supplementary Fig. 7b), further confirming its good structural stability.

Additionally, the FCNSs could maintain stable Poisson’s ratios nearly close to 0 (–0.1 to 0) during the high compression and recovery process (maximum *ε* of 80%) (Supplementary Figs. 7c and 9), which was lower than most existing cellular foams (>0.2)^46,47^ (see details in Supplementary Methods). Furthermore, the FCNS70 also showed remarkable dynamic mechanical stability (Supplementary Fig. 7d), the storage modulus, loss modulus, and damping ratio of the FCNS70 nearly remained stable when the compression frequency increased from 0.1 to 10 Hz, indicating the good resilience performance of the FCNSs^48^. To gain insight into the good mechanical properties of FCNSs, we observed the deformation behavior of the sponge cavity wall under the action of external force by in situ SEM. As shown in Fig. 2h, i, when the compressive strain increased from 0 to 60%, the lamellar cell walls and interlamellar SNFs were gradually bent and buckled, respectively. Interestingly, the rGO networks and SNFs were not found to break during compression. Subsequently, the curved cell walls and SNFs completely returned to their initial position after the strain was eliminated, further highlighting the robust structure of the FCNS70^38,49,50^. Besides, Fig. 2g showed the relationship between the relative Young’s modulus (*E*/*E*_s_) of various materials and the relative bulk density (*ρ*/*ρ*_s_). Different from the low-stress transfer efficiency of random structural inorganic foams (the relation index of *E*/*E*_s_ versus *ρ*/*ρ*_s_ > 2.5)^20,45,51–53^, the relationship of the FCNSs was *E*/*E*_s_ ~ *ρ*/*ρ*_s_^0.7^ (see details in Supplementary Methods), indicating that the FCNSs had similar elastic behavior as the low-density open-cell structured materials^54^; meanwhile, the lamellar cavity wall of FCNSs could be bent reversibly under the action of external force, so the loading stress could be effectively transferred among the cavity walls of the sponges. More interestingly, compared with the little tensile performance of existing ceramic fibrous sponges, the unique hierarchically entangled structures also endowed FCNS70 with certain stretchable properties (Supplementary Fig. 8b). The tensile stress first improved linearly with the increase of tensile strain, and Young’s modulus reaching 282 kPa. Further increase in tensile strain induced the plastic extension of the cell walls and resulted in the gradual fracture of the FCNSs; meanwhile, the tensile stress and strain of FCNS70 were 12.56 kPa and 4.8%, respectively^20,49^.

**Fig. 3:**
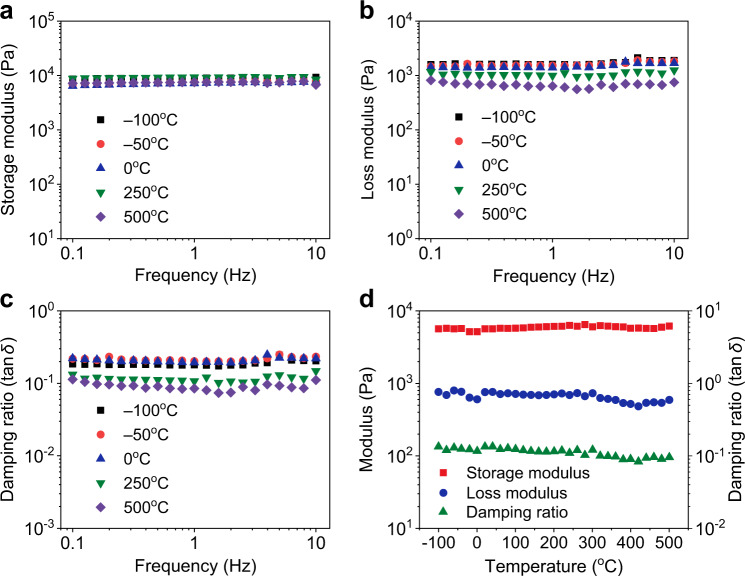
Mechanical properties of the FCNSs over a wide range of temperatures. **a**–**c** Storage modulus, loss modulus, and damping ratio of the FCNS70 versus frequency (0.1–10 Hz) at temperatures from −100 to 500 °C. **d** The temperature dependence of the storage modulus, loss modulus, and damping ratio for FCNS70.

Current sound-absorbing materials are mainly made of polymer, which has good elasticity at room temperature but cannot use at high temperatures. In contrast, ceramic materials possess good heat resistance but suffer from stubborn brittleness with little deformation. Our FCNSs combined the structural characteristics of polymer materials and the high-temperature resistance of ceramics, which still exhibited good elasticity at high temperatures. As shown in Fig. 3a–c, the FCNS70 exhibited stable viscoelasticity at different ambient temperatures, the storage modulus, loss modulus, and damping ratio almost remained at stable values from −100 to 500 °C^55^. Moreover, the relationship of dynamic mechanical performances of the FCNSs versus continuously changing temperature was further investigated, as shown in Fig. 3d. The storage modulus, loss modulus, and damping ratio of the FCNS70 almost remained unchanged with the ambient temperature continuously changing from −100 to 500 °C, indicating that the FCNSs can be used for noise absorption in extreme temperatures^56^. Evidence of good thermal endurance of FCNS70 also came from thermogravimetry analysis (TGA), as shown in Supplementary Fig. 10; the TGA plot of FCNSs only showed a significant weight loss when the temperature was >500 °C. Additionally, no structural collapse and ignition were observed when the FCNSs were compressed in a high-temperature flame (~550 °C) with a large strain (Supplementary Fig. 11), highlighting that the FCNSs still had superelasticity at high temperature. Besides, the FCNSs almost had no plastic deformation after being compressed in liquid nitrogen (–196 °C) with a large strain of 80% (Supplementary Fig. 12 and Supplementary Movie 3), confirming superelasticity of FCNSs under low temperature.

### Sound absorption properties of FCNSs

**Fig. 4:**
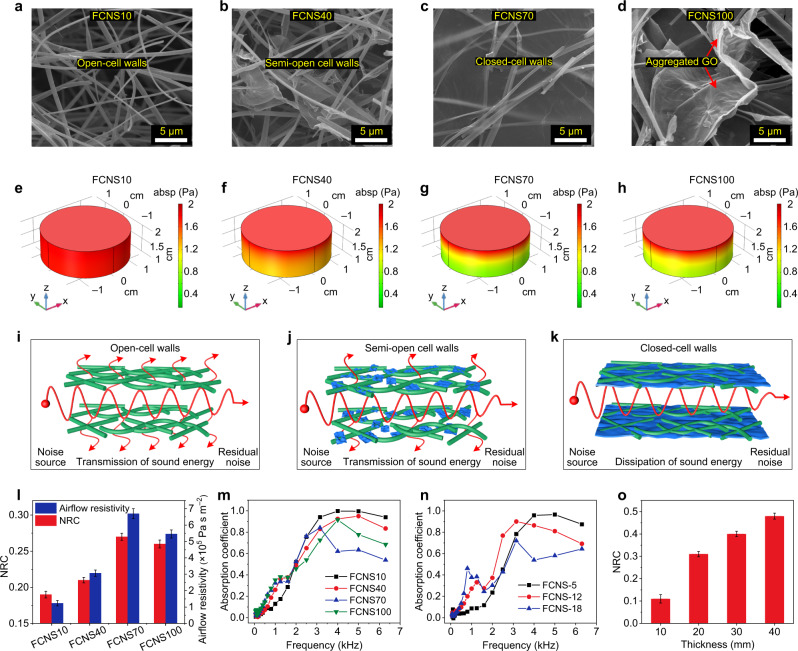
Sound absorption properties of FCNSs. SEM images showing the cell walls of **a** FCNS10, **b** FCNS40, **c** FCNS70, and **d** FCNS100. **e**–**h** The corresponding absolute sound pressure distribution of various FCNSs. Schematic illustrations of the sound absorption mechanism for **i** open-cell walls, **j** semi-open cell walls, and **k** closed-cell walls. **l** Influences of GO loading amount on NRC and airflow resistance of the FCNSs. **m** Variation of the absorption coefficient of the relevant FCNSs. **n** Effects of density on the absorption coefficient of FCNSs. **o** NRC of FCNS-5 with various thicknesses. Error bars in **l**, **o** represent the standard deviations of three replicates.

Taking into consideration the hierarchically entangled structures, desired mechanical properties, and good temperature resistance, the FCNSs showed great application potential in noise absorption. In view of the porous structure characteristics of sponges, the widely used Johnson–Champoux–Allard (JCA) model was applied to guide and optimize the design of FCNSs^27,57^. Based on the JCA theoretical model, we have explored the influence of acoustic parameters (such as volume density, thickness, and airflow resistivity) on noise absorption properties of FCNSs. Then, feedback designs on the structural parameters of FCNSs were further carried out to achieve broadband noise absorption (see details in Supplementary Methods and Supplementary Discussions). Generally, the noise absorption performances were decided by the assembly structure, which was mainly influenced by the mass ratio of SNF/GO (from 10/1 to 10/10). As presented in Fig. 4a–d, the coverage area of rGO networks on nanofibrous cell walls gradually increased with the improvement of GO loading amounts, resulting in the reduction of connectivity of cell walls within FCNSs. Moreover, the airflow resistance of FCNSs was greatly improved from 1.2 × 10^5^ to 6.7 × 10^5^ Pa s m^−2^ with increasing mass ratios of SNF/GO (from 10/1 to 10/7), indicating that the GO networks effectively optimized the pore structure of the FCNSs (Fig. 4l). However, further increasing GO loading amount decreased the airflow resistance, which was because that excess GO agglomerated and nonuniformly distributed caused by the strong π–π attraction (Fig. 4d). Furthermore, noise absorption properties are generally appraised by the NRC of materials, which is the average value of absorption coefficients in 250, 500, 1000, and 2000 Hz^11,12,27^. The NRC of the FCNSs with increasing GO loading amounts were 0.19, 0.21, 0.27, and 0.26, respectively (Fig. 4l, m), which was consistent with flow resistance.

To explore the sound absorption mechanism, 3D structural models of FCNSs were constructed, and the absolute sound pressure in typical 500 Hz of the FCNSs were calculated by the COMSOL software (see Fig. 4e–h and Supplementary Methods). Obviously, the sound pressure field models using color as scale showed that the sound pressure drop of the FCNS70 (Fig. 4g, red to green color) was greater than that of the FCNS100 (Fig. 4h, red to light green color) and FCNS40 (Fig. 4f, red to light yellow color), and much greater than FCNS10 (Fig. 4e, red color), which was consistent with NRC. Based on the above results, we established a series of sound absorption diagrams of open cell walls, semi-open cell walls and closed-cell walls. As shown in Fig. 4i–k and Supplementary Fig. 13, the open-cell walls had a highly connected nanofibrous network structure and lower airflow resistance, so sound waves could easily transmit and penetrate through the sponges, and the sound energy loss was relatively small (Fig. 4i). By contrast, some assembled GO networks could randomly block the hole in the fibrous cell walls, thus effectively reducing the sound wave transmission through the cell walls (Fig. 4j). Furthermore, the closed-cell walls could completely prevent the transmission of sound waves and make the dissipation path much longer, thus achieving effective energy dissipation (Fig. 4k).

Additionally, the influence of density on the noise absorption performances of the FCNSs (thickness of 10 mm) was shown in Fig. 4n. The low-density FCNS-5 possessed poor low-frequency (<1000 Hz) absorption and good high-frequency (>1000 Hz) absorption, which was due to the constant friction and dissipation between high-frequency sound waves and the macropore structure of FCNSs^58^. On the contrary, the high-density FCNS-18 possessed good low-frequency absorption but poor high-frequency absorption, which was because that the small porous structure increased dissipation probability of low-frequency waves, but high-frequency sound waves that feature with lower penetration were reflected when entering FCNS-18^59^. Moreover, we further studied the effects of thickness on the sound absorption performance of the FCNSs; as shown in Fig. 4o and Supplementary Fig. 14, the absorption coefficient and NRC were improved with the increase of thickness, which was attributed to increasing dissipation path^27^.

### Sound absorption properties of sandwiched FCNSs

**Fig. 5:**
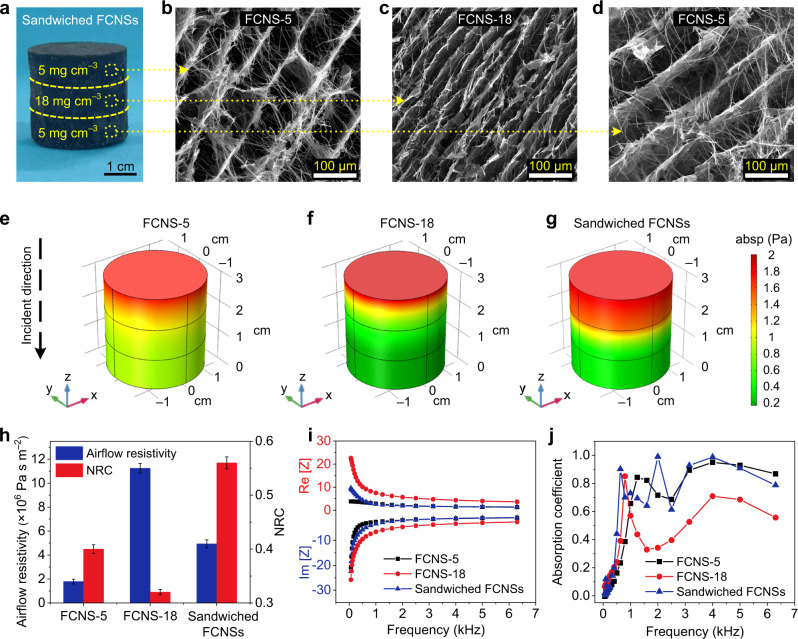
Sound absorption properties of sandwiched FCNSs. **a** Optical and **b**–**d** SEM images of sandwiched FCNSs showing the macroscopic continuity and fluffy–dense–fluffy microscopic structure. Absolute sound pressure distribution of **e** FCNS-5, **f** FCNS-18, and **g** sandwiched FCNSs in 500 Hz. **h** Airflow resistivity and NRC of FCNS-5, FCNS-18, and sandwiched FCNSs. Error bars represent the standard deviations of three replicates. **i** The specific surface acoustic impedance and **j** sound absorption coefficients of FCNS-5, FCNS-18, and sandwiched FCNSs.

Inspired by the fascinating effects of density and thickness on the sound absorption coefficient of the FCNSs, we further constructed the sandwiched architecture in the thickness direction of the FCNSs (Supplementary Methods). As shown in Fig. 5a–d, the optical paragraph and SEM images derived from three layers showed the continuous structure of sandwiched FCNSs and the gradually changed pore size with variant density from 5 to 18 and 5 mg cm^−3^ (see details in Supplementary Methods). Surprisingly, no much difference was found in mechanical properties between the sandwiched FCNSs and their individual layers (only FCNS-5 or FCNS-18), which was attributed to the stable entangled structures comprised by GO networks and SNFs (Supplementary Discussions). Simultaneously, the airflow resistance of the sandwiched FCNSs (4.9 × 10^6^ Pa s m^−2^) was between FCNS-5 (1.8 × 10^6^ Pa s m^−2^) and FCNS-18 (11.3 × 10^6^ Pa s m^−2^) (Fig. 5h), which was proportional to the sample density^60,61^. Moreover, the corresponding specific acoustic impedance had also been calculated, where the real (Re [Z]) and imaginary (Im [Z]) parts represented the transmission resistance and inertial acoustic resistance of sound waves, respectively (see Fig. 5i and Supplementary Methods). The transmission resistance of sandwiched FCNSs was between FCNS-5 and FCNS-18 in the low-frequency (<1000 Hz) range, and it was consistent with FCNS-5 in the high-frequency (>1000 Hz) range; while the acoustic resistance was in a similar trend^11,62^. Based on the above acoustic parameters, the contribution of the sandwich structure to the sound absorption performances was further estimated, as shown in Fig. 5j. The FCNS-5 and FCNS-18 showed good high-frequency and low-frequency sound absorption, respectively. Notably, the absorption coefficient of sandwiched FCNSs always outperformed that of the FCNS-5 and FCNS-18 in almost all frequency bands from 63 to 6300 Hz^12,27^ (see Supplementary Methods), and the NRC of sandwiched FCNSs achieved 0.56, satisfying the requirement of high-efficiency sound absorption materials (NRC ≥ 0.56)^63^, whereas the values for FCNS-5 and FCNS-18 were only 0.40 and 0.32, respectively. Additionally, the design values of sound absorption coefficients of sandwiched FCNSs were also calculated based on the above-mentioned JCA module, and a detailed comparison between experimental values and designed values is described in Supplementary Discussions.

**Fig. 6:**
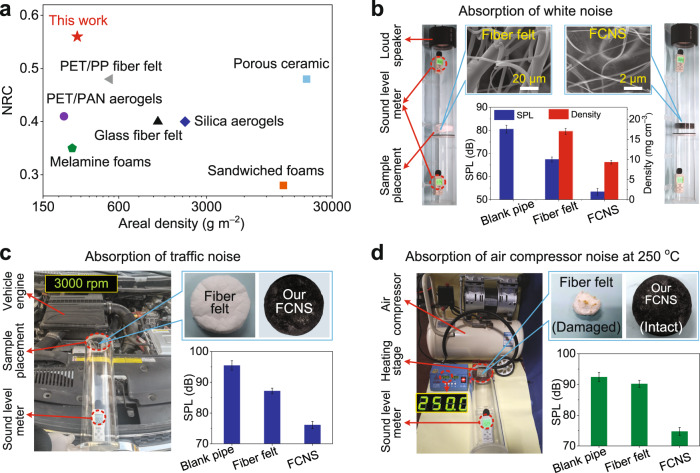
Application performances of sandwiched FCNSs. **a** Comparison of NRC for the different noise reduction materials and the sandwiched FCNSs. Photographs showing the direct application of the commercial fiber felt and sandwiched FCNSs on the **b** white noise absorption, **c** automobile engine noise absorption at 3000 rpm, and **d** air compressor noise absorption at the high temperature of 250 °C. Error bars in **b**–**d** represent the standard deviations of three replicates.

To explore the sound absorption mechanism of the sandwich structure, the 3D structural models of FCNS-5, FCNS-18, and sandwiched FCNSs were constructed based on the acoustic characteristic parameters^60^ (Supplementary Table 5), as illustrated in Supplementary Methods. Obviously, the sound pressure drops of the FCNS-18 and sandwiched FCNSs (red to dark green color) were much greater than FCNS-5 (red to yellow-green color), which was consistent with the poor low-frequency absorption of FCNS-5 (Fig. 5e–g and Supplementary Fig. 15). However, in contrast to the rapid drop in sound pressure of FCNS-18 as a consequence of its overlarge density and excessive reflections, the sound pressure of the sandwiched FCNSs was generally high in the first unit and then decreased continuously, indicating that more sound waves could enter and then were gradually dissipated, which were consistent with high absorption coefficients of sandwiched FCNSs. Based on the above results, we further constructed the structural element models and simulated sound absorption mechanisms (Supplementary Fig. 16), the low-density FCNS-5 with macroporous structure preferred to dissipating high-frequency noise (Supplementary Fig. 16a), while the high-density FCNS-18 with a small porous structure preferred to dissipating low-frequency noise (Supplementary Fig. 16b). Interestingly, by designing a sandwiched architecture, an attractive broadband sound absorption behavior was found: the low-density and high-density lamellar structures were responsible for effectively dissipating high-frequency and low-frequency sound waves, respectively, thus successfully absorbing the broadband sound waves (Supplementary Fig. 16c). Besides, the sandwich structure effectively increased the internal interface of FCNSs, which enhanced the multistage reflection path of sound waves inside the material and thus successfully increased the sound energy dissipation^9^. To confirm the noise reduction performance of the sandwiched FCNSs, its NRC and corresponding areal density (Supplementary Discussions) were compared with typical already reported and commercial noise reduction materials^13,27,39,40^ (Supplementary Table 1 and Supplementary Discussions). As shown in Fig. 6a, the sandwiched FCNSs exhibited high noise absorption (NRC of 0.56) with ultralight property (280.8 g m^−2^) in contrast to the uniform structures of present noise absorbers, which was because that the unique hierarchical structure increased the contact area of sound waves, providing more frictional resistance for the acoustic energy. Meanwhile, the resonance of the cavity walls of the sandwiched FCNSs also dissipated a small part of acoustic energy^8^ (see details in Supplementary Discussions).

As a proof of concept for traffic noise reduction applications, the specific sound attenuation capability of the sandwiched FCNSs was further evaluated, which was mainly determined by the imaginary part of the sound wave propagation constant^64^, thus depending on acoustic characteristic parameters and thickness (see details in Supplementary Methods). As shown Fig. 6b, the white noise of ~80 dB was generated by a loudspeaker within a well-sealed pipe to mimic traffic noise, after passing commercial fiber felt at the middle of the pipe, the noise only decreased to 67.4 dB due to the large fiber diameter of >5 μm, the large density of 17 mg cm^−3^, and open-cell walls. In dramatic contrast, our sandwiched FCNSs could sharply decrease noise to 53.4 dB by their closed-cell walls, highlighting the great practical application performance. We further monitored the attenuation capacity of real automobile engines noise, as shown in Fig. 6c and Supplementary Fig. 17a. A bottom sealed round pipe loading with commercial fiber felt and our sandwiched FCNSs were placed next to a working engine, and the sandwiched FCNSs reduced noise by 19.4 dB (from 95.5 to 76.1 dB), whereas the fiber felt only reduced by 8.3 dB (from 95.5 to 87.2 dB). Moreover, the sandwiched FCNSs exhibited promising noise attenuation against running air compressor in high temperature, as shown in Supplementary Fig. 17b, the sandwiched FCNSs reduced noise by 19 dB (from 94.2 to 75.2 dB), whereas the commercial fiber felt only reduced by 10.1 dB (from 94.2 to 84.1 dB). Furthermore, when a heating stage with the temperature of 250 °C was close to two samples, the sandwiched FCNSs not only reduced noise by 17.7 dB after 10 min but also maintained intact morphology; whereas the fiber felt was significantly decomposed and shrunk, with almost no sound attenuation (from 92.4 to 90.2 dB) (Fig. 6d and Supplementary Fig. 17c). These results indicate that the sandwiched FCNSs could serve as robust noise-absorbing materials with potentially broad applications, such as traffic noise reduction, industrial noise reduction, and domestic noise reduction, especially for safety noise reduction in high-temperature environments.

## Discussion

The design and construction of FCNSs open a path for searching the advanced applications of 1D ceramic nanofibers in 3D macrostructures. In this study, FCNSs served as a practical verification of the conceptual model; in consideration of the rich source of ceramic material as well as the facile preparation of FCNSs, our research will provide ideas for ceramic nanofibrous sponges for application in various fields. For example, bioceramics (such as ZrO_2_ and hydroxyapatite) have been widely used as bone tissue substitutes; thus, creating 3D FCNSs using these bioceramic nanofibers can more really simulate bone tissue by providing suitable direction for cell growth^65^. Moreover, honeycomb ceramics (such as Al2O_3_ and TiO_2_) are usually used as catalyst carriers but suffer from stubborn brittleness; the FCNSs not only possess robust flexibility but also enable to load of more nano-catalysts, which could significantly improve the application performance. Additionally, some functional materials (such as Au, Fe_3_O_4_, and other nanoparticles) could be easily embedded in the open space of FCNSs, enabling the fabrication of multifunctional composite sponges.

In summary, we presented a robust methodology to construct FCNSs with hierarchically entangled graphene networks. Benefiting from the entangled structure composed of rGO network-wrapped ceramic nanofibers, the FCNSs achieved ultralight properties, temperature-invariant superelasticity (4.3% plastic deformation at the 1000th cycle), and good bendability (unchanged structures over 1000 times). Furthermore, the hierarchically entangled sandwich structures enabled a versatile multi-interface reflection capability, contributing to the enhanced sound absorption performance (NRC of 0.56). Moreover, the comprehensive advantages of ceramic and rGO endowed FCNSs with good heat resistance and enhanced moisture insulation. We envision that these exceptional sandwiched FCNSs that can be easily scaled up will pave the way for efficient sound-absorbing materials used for thoroughly removing noise, involving traffic noise reduction, industrial noise reduction, and domestic noise reduction.

## Methods

### Fabrication of the FCNSs

In the typical process for preparing the FCNS70 with a density of 8 mg cm^−3^, 0.67 g of GO powder was dispersed in 66.3 g of ultrapure water and sonicated for 4 h at an ultrasonic temperature of 25 ± 5 °C to get the GO dispersion with the content of 1 wt%. Then, 0.96 g of flexible SNF membrane was cut into pieces and placed in a 67 g of aforementioned GO dispersion by a high-speed homogenizer (13,000 rpm and 20 min) to obtain a homogeneous and stable GO/nanofiber dispersion. Subsequently, 2.68 g of ascorbic acid (*m*_ascorbic acid_:*m*_GO_ = 4/1) was added to the nanofiber/GO dispersion for further homogenization for 20 min. The dispersion was then poured into the pre-prepared mold, controlling the flow rate of liquid nitrogen just covering the bottom of the mold to obtain a directional frozen block. This was followed by transferring the frozen block to a lyophilizer for vacuum freeze-drying for 16 h obtained the GO/SNF sponges. During the preparation process of sponges, the ambient temperature and humidity were maintained at 25 ± 2 °C and 45 ± 5%, respectively. Afterward, the GO/SNF sponges were heated at 90 °C for 6 h to obtain hydrophobic FCNSs. Besides, various FCNSs with a wide range of densities from 2 to 20 mg cm^−3^ were obtained by controlling the concentrations of the precursor GO/SNFs dispersions.

### Fabrication of sandwiched FCNSs

First, the GO/nanofiber dispersion of FCNS-5 was frozen in a pre-prepared mold according to the above method; after it was completely frozen, the dispersion of FCNS-18 was added immediately for further freezing. When the dispersion of FCNS-18 changed from liquid to solid, the upper edge of the FCNS-5 frozen block was slightly thawed, thus mixing well with the dispersion of FCNS-18, and then the interface was frozen again during the dispersion of FCNS-18 freezing. Subsequently, another dispersion of FCNS-5 was added on top of the FCNS-18/FCNS-5 frozen block and frozen by the same method. Therefore, there was no obvious interface in the sandwiched frozen block, which ensured good structural continuity. Afterward, the composite frozen blocks were freeze-dried and heated at 90 °C for 6 h to obtain sandwiched FCNSs.

### Characterization

The morphology and microstructures of the sponges were characterized using SEM (VEGA 3, TESCAN Ltd., Czech Republic). Elemental mapping was recorded using EDS (Bruker Quantax 400) spectroscopy attached to SEM. FTIR spectroscopic analysis was investigated by Nicolet iS10 Spectrometer (Thermo Fisher Scientific Inc., USA). The crystallinity of the FCNSs was tested by XRD (D8 Advance, Bruker, German) with a Cu radiation source (*λ* = 1.5406 Å). Raman spectra were taken on a Raman spectrometer (LabRAM HR Evolution, HORIBA Ltd., France) with 532 nm laser excitation. The diameters of SNFs were obtained by the measuring tool of Adobe Acrobat 9 Pro. The compressive and buckling performances were evaluated by a dynamic thermal mechanical analyzer (TA-Q850, TA Instruments, USA) equipped with corresponding compression and buckling clamps. During the compressive test process, cylindrical sponges with a diameter of 20 mm were prepared, and the strain rate was maintained as 100 mm min^−1^. During the buckling test process, rectangular sponges with the size of 22 × 6 × 3 mm^3^ were prepared, and the strain rate was maintained as 20 mm min^−1^. The tensile performances of sponges were measured by the fiber tensile tester (XQ-1C, Shanghai New Fiber Instrument Co, Ltd, China). The water contact angle was detected by a contact angle goniometer (Kino SL200B, USA), and the volume of a water droplet was controlled in 3 μL.

The flow resistance of the FCNSs was tested by a flow resistance tester (Autoneum Management Co., Ltd, Switzerland) based on the ISO 9053 standard, where the diameter of the samples was 100 mm, and the airflow rate of controlled air was 0.0005 m s^−1^. The noise reduction performance of obtained sponges was evaluated using the impedance tube (SW422 and SW477, BSWA Technology Co., Ltd, China). A large tube (diameter of 10 cm) and a small tube (diameter of 3 cm) were used to measure the frequency ranges of 63–1600 and 1000–6300 Hz, and the cylindrical FCNSs with a diameter of 10 and 3 cm were prepared for measurement, respectively. Based on relevant test methods and research significance, the noise absorption test was in the range of 63–6300 Hz^12^. The sound absorption coefficients for 1/3 octave band frequencies (63, 80, 100, 125, 160, 200, 250, 315, 400, 500, 630, 800, 1000, 1250, 1600, 2000, 2500, 3150, 4000, 5000, and 6300 Hz) were taken, and the results were plotted into absorption coefficient curves. Each sample was tested five times to minimize the error. All the tests of sound absorption coefficients were performed at a temperature of 25 ± 2 °C and humidity of 45 ± 5%.

## Supplementary information

Supplementary Information

Description of Additional Supplementary Files

Supplementary Movie 1

Supplementary Movie 2

Supplementary Movie 3

## Data Availability

The experimental data that support the findings of this study are available from the corresponding author upon reasonable request.
