# Supplementary material for: Flexible ceramic nanofibrous sponges with hierarchically entangled graphene networks enable noise absorption
Source: Nat Commun. 2021 Nov 15;12:6599. doi: 10.1038/s41467-021-26890-9 (PMC8593031; doi:10.1038/s41467-021-26890-9)
Supplement: Supplementary file 1 — Supplementary Information [file 41467_2021_26890_MOESM1_ESM.pdf]

Flexible Ceramic Nanofibrous Sponges with Hierarchically Entangled Graphene Networks Enable Noise Absorption

Zong *et al.*

Supporting Information contains:

Supplementary Notes

Supplementary Figures 1 – 23

Supplementary Tables 1 – 5

Supplementary Methods

Supplementary Discussions

Supplementary References

Supplementary Notes

Nomenclature

ρ_a	areal density	Λ	viscous characteristic length
ρ	volume density	Λ'	thermal characteristic length
t	thickness	ΔP	pressure difference between two sides of sponge
ρ_{eq}	equivalent density		
K_{eq}	equivalent bulk modulus	ρ_s	bulk density of the material
η	viscosity of air	S_1	cross-section area of the sponge
ρ_0	density of air	S_2	surface area of the sponge
γ	specific heat ratio of air	U	volume velocity of airflow
ω	angular frequency	c	cross-sectional shape factor
P_r	Prandtl number	k_r	real part of k
P_0	atmospheric pressure	k_i	imaginary part of k
i	imaginary unit	L	attenuation
Z_c	characteristic impedance	E	Young's modulus
k	propagation constant	A	constant of geometric proportionality
Z_s	surface impedance	n	power exponent
α	sound absorption coefficient	E_S	bulk modulus
c_0	sound velocity of air	v	Poisson's ratio
φ	porosity	ε_x	transverse strain
α_∞	tortuosity factor	ε_y	longitudinal strain
σ	airflow resistivity	S_g	specific surface area
V	volume	m	mass

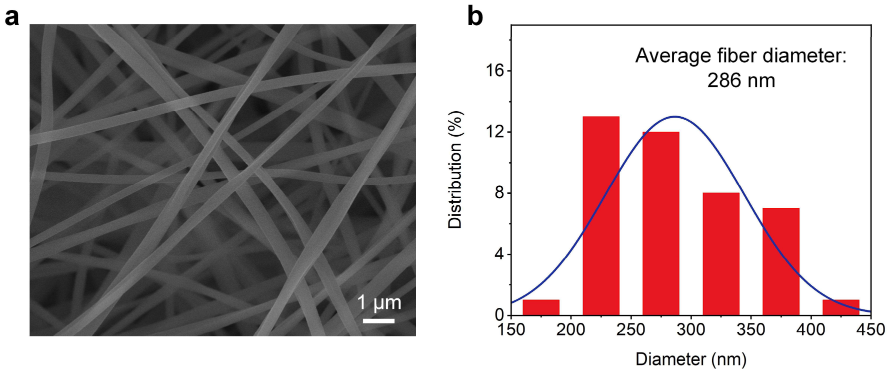

Supplementary Fig. 1 (**a**) SEM image of SiO_2 nanofibers. (**b**) Histogram showing the fiber diameter distribution of SiO_2 nanofibers.

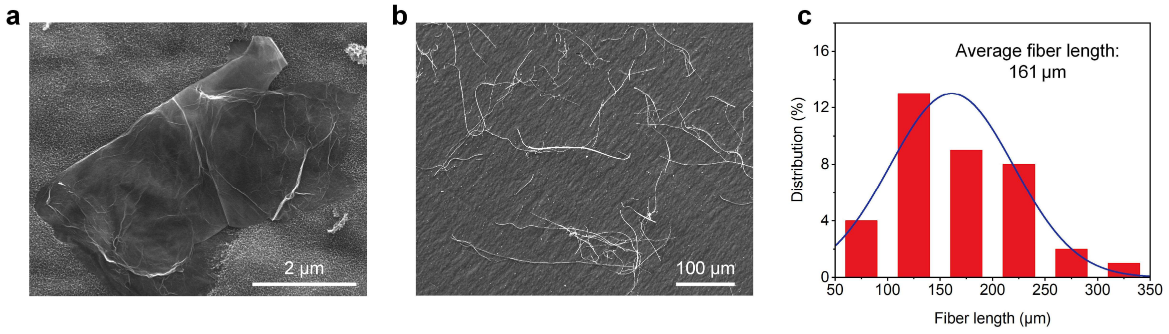

Supplementary Fig. 2 (**a**) SEM image of GO. (**b**) SEM image of the homogenized SiO_2 nanofibers. (**c**) Histogram showing fiber length distribution of the homogenized SiO_2 nanofibers.

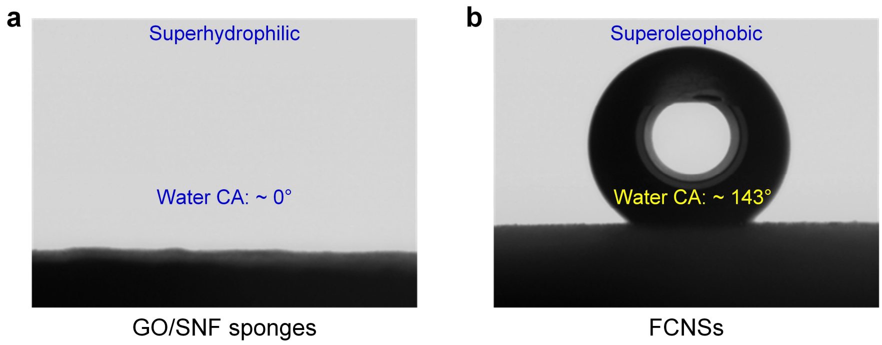

Supplementary Fig. 3 Wettability of the samples before and after reduction: (**a**) GO/SNF sponges and (**b**) FCNSs.

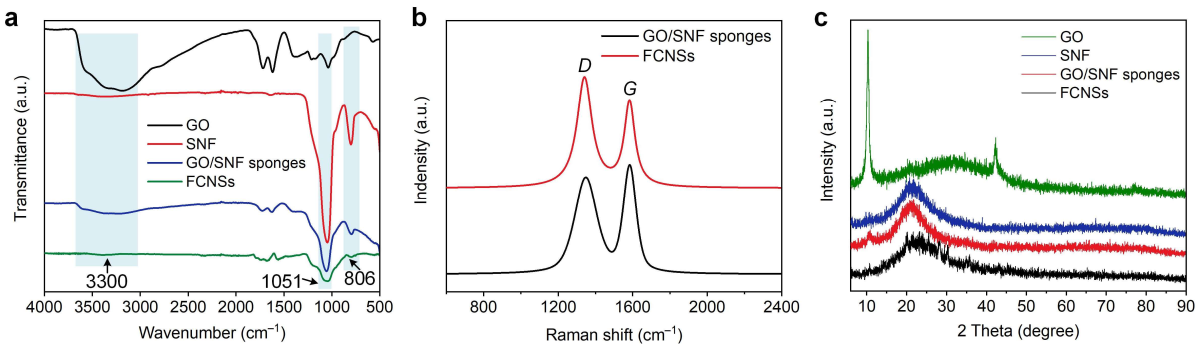

Supplementary Fig. 4 (**a**) FTIR spectra of GO, SNF, GO/SNF sponges and FCNSs. (**b**) Raman spectra of GO/SNF sponges and FCNSs. (**c**) XRD spectra of GO, SNF, GO/SNF sponges and FCNSs.

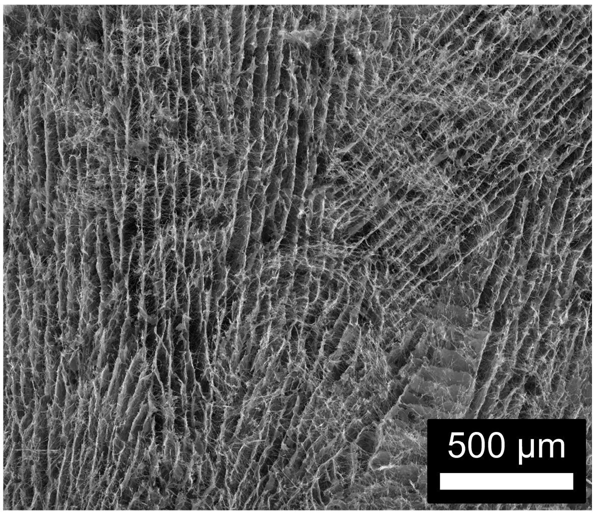

Supplementary Fig. 5 SEM image shows the micro-orientation structure of the FCNSs.

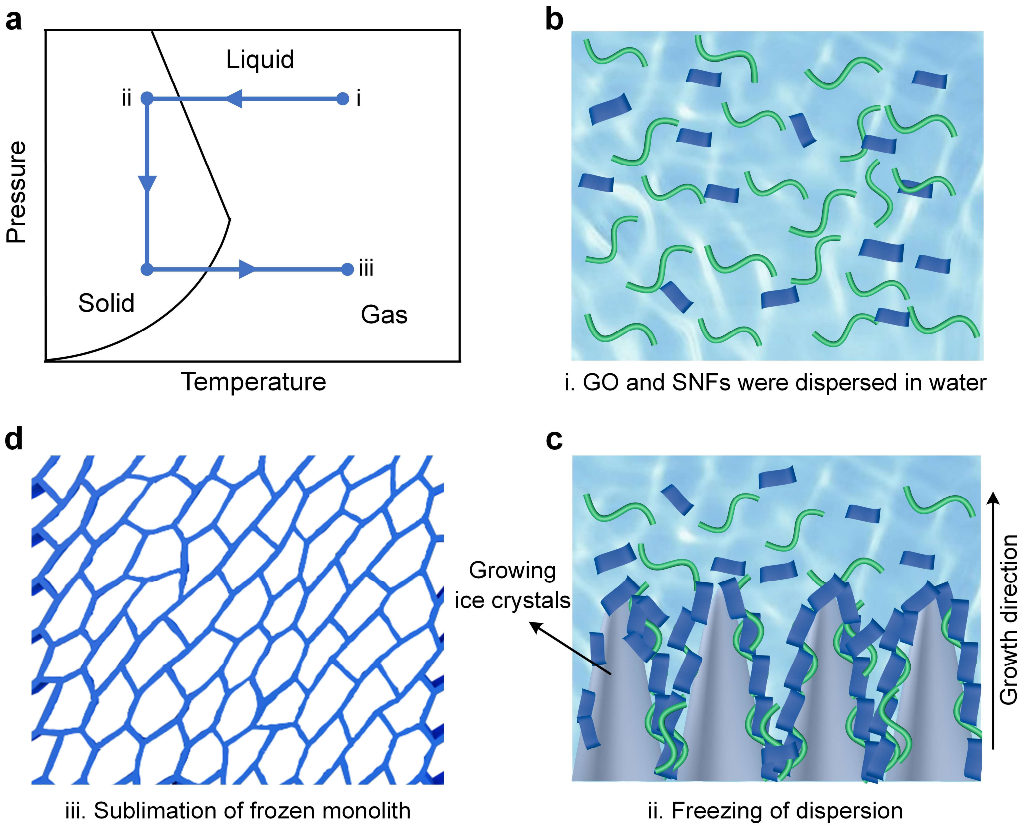

Supplementary Fig. 6 (**a**) Phase diagram of the three processing steps of freeze-shaping. (**b-d**) Schematical illustration of the formation principles for the hierarchically entangled structures.

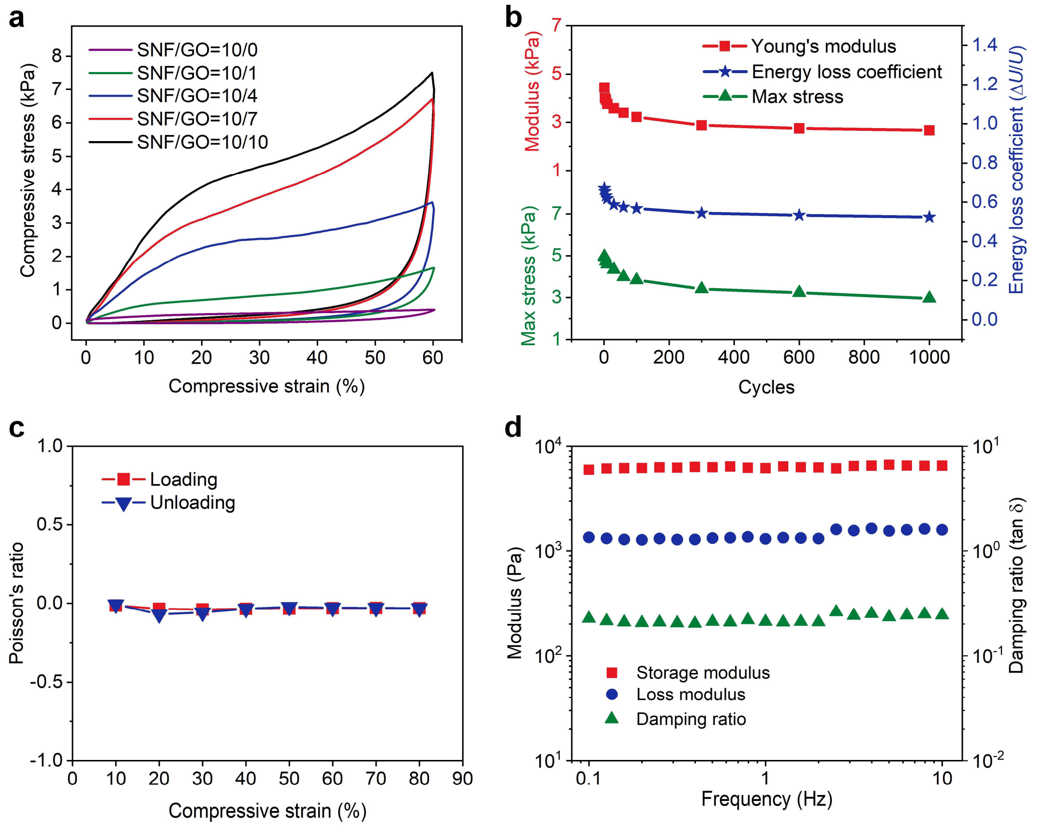

Supplementary Fig. 7 (**a**) Compression performance of the FCNSs with various ratios of SNF/GO. (**b**) Young's modulus, energy loss coefficient and max stress versus compressive cycles. (**c**) The Poisson's ratio of the FCNSs versus strain. (**d**) The frequency dependence of the storage modulus, loss modulus and damping ratio for FCNSs.

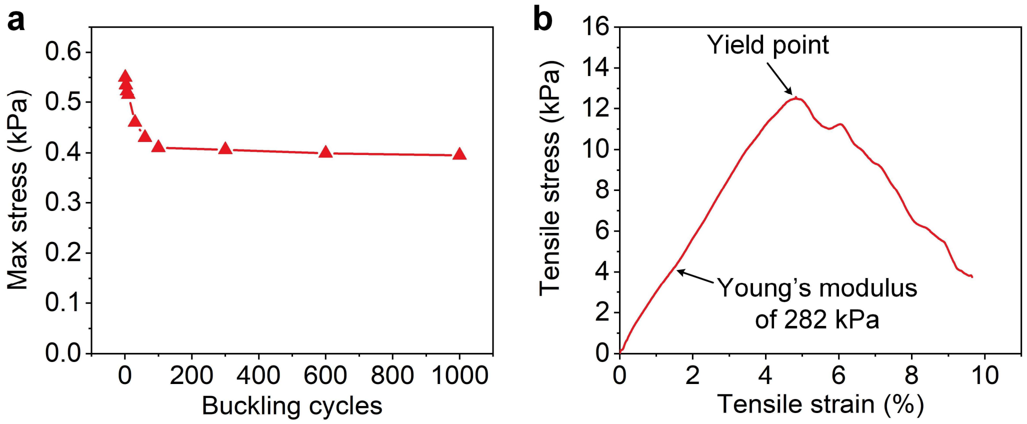

Supplementary Fig. 8 (**a**) Buckling stress versus buckling cycles. (**b**) The tensile σ-ε curve for the FCNS70.

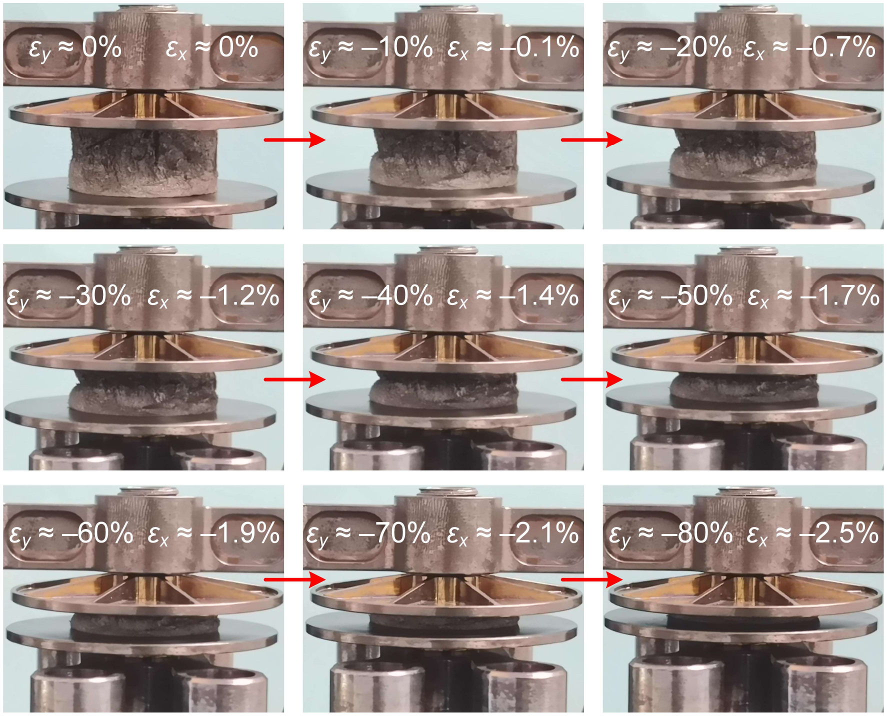

Supplementary Fig. 9 Optical images showing the concave deformation of the FCNSs during the compressive process with increased strain, illustrating the negative Poisson's ratio of the FCNSs.

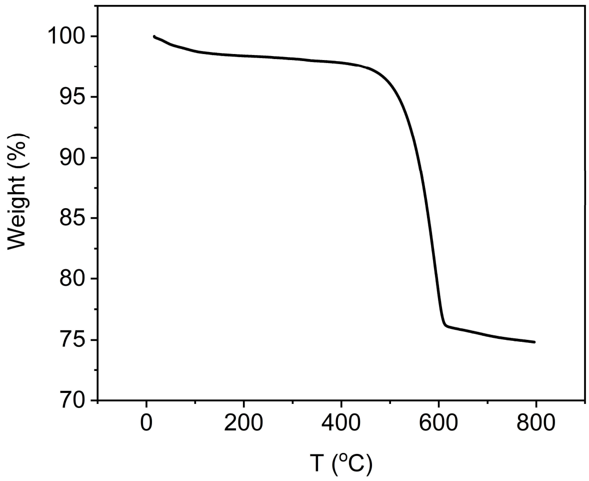

Supplementary Fig. 10 TGA analysis of FCNSs.

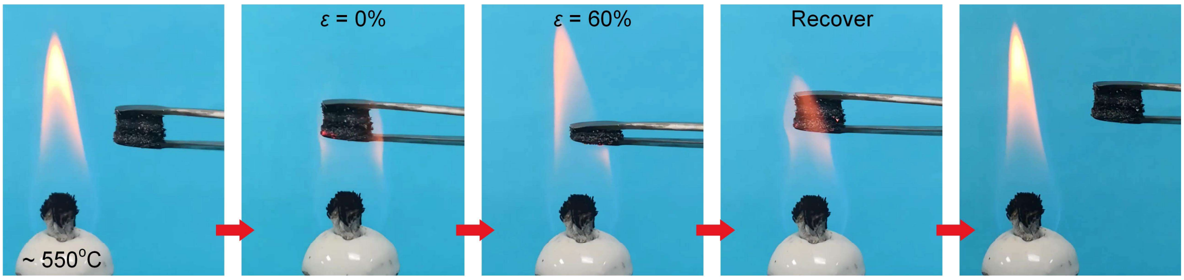

Supplementary Fig. 11 Digital photographs of the burning test showing the high-temperature stability of the FCNSs in the flame of an alcohol lamp.

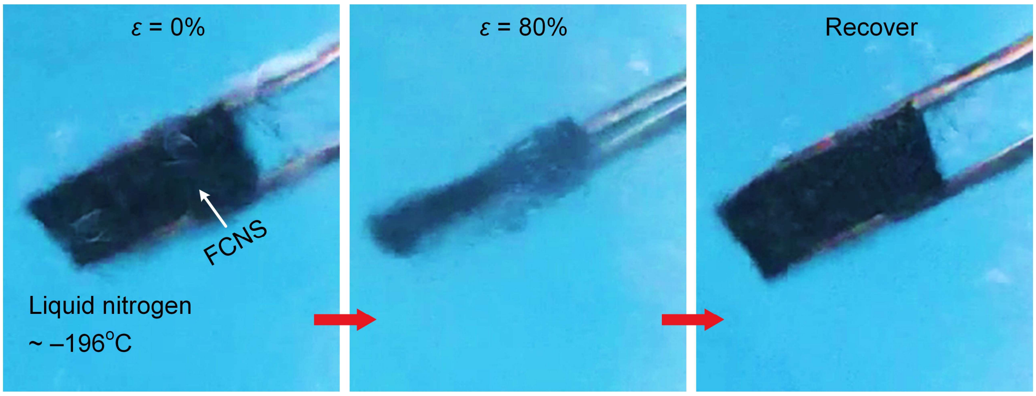

Supplementary Fig. 12 Optical images showing the elastic resilience of an FCNS in the liquid nitrogen (–196 °C).

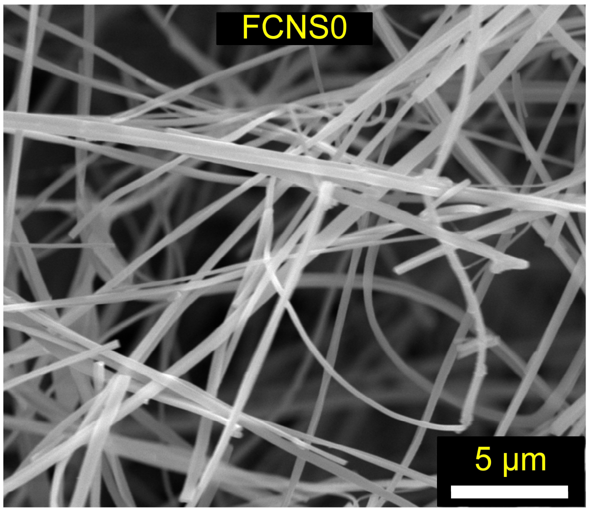

Supplementary Fig. 13 SEM image showing the cell wall of FCNSs without GO.

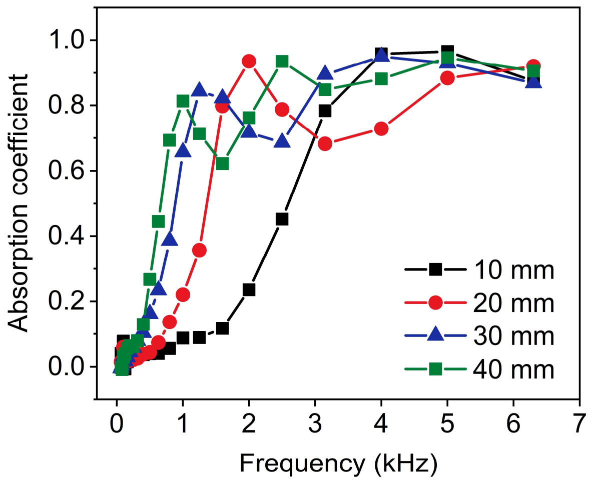

Supplementary Fig. 14 Sound absorption coefficients of FCNS-5 with various thicknesses.

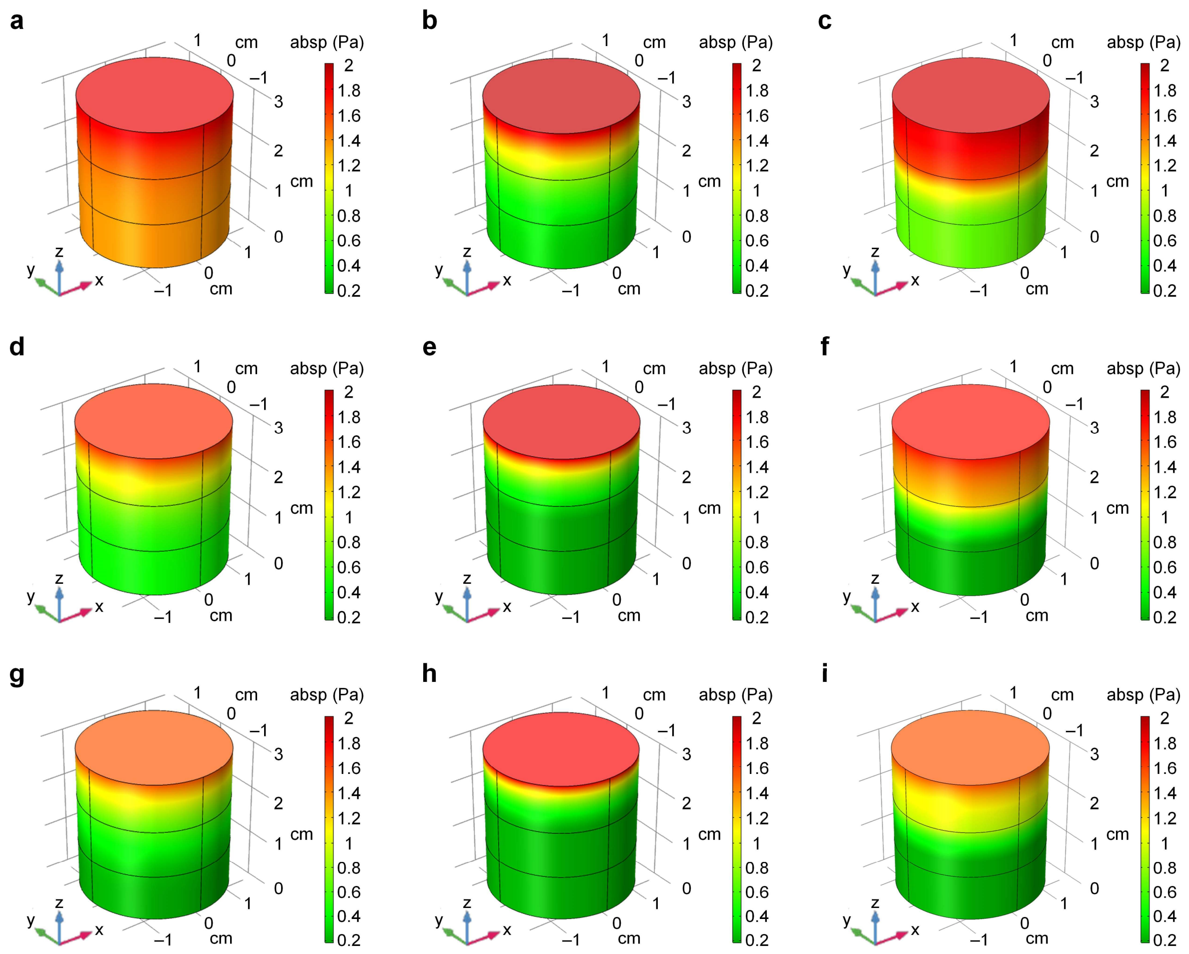

Supplementary Fig. 15 Absolute sound pressure distribution of (**a**, **d**, **g**) FCNS-5, **(b**, **e**, **h**) FCNS-18 and (**c**, **f**, **i**) sandwiched FCNSs in 250, 1000 and 2000 Hz, respectively.

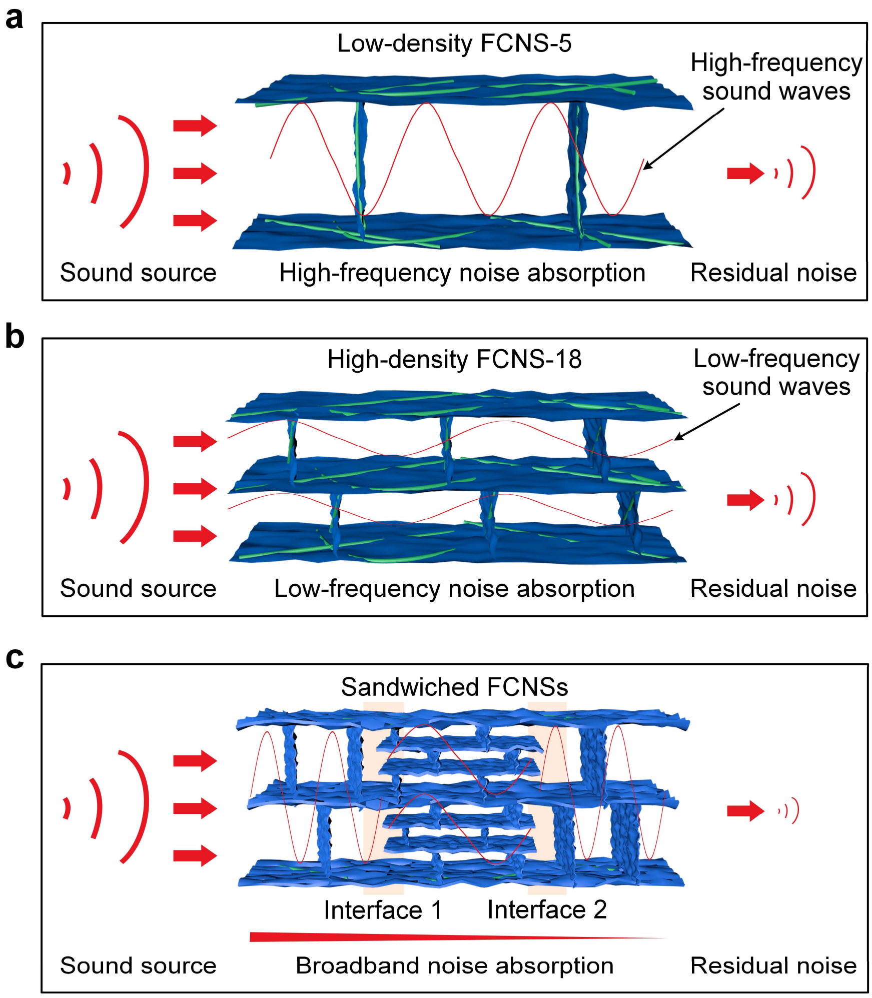

Supplementary Fig. 16 Schematic illustrations of the mechanism for the sound absorption of (**a**) FCNS-5, (**b**) FCNS-18 and (**c**) sandwiched FCNSs

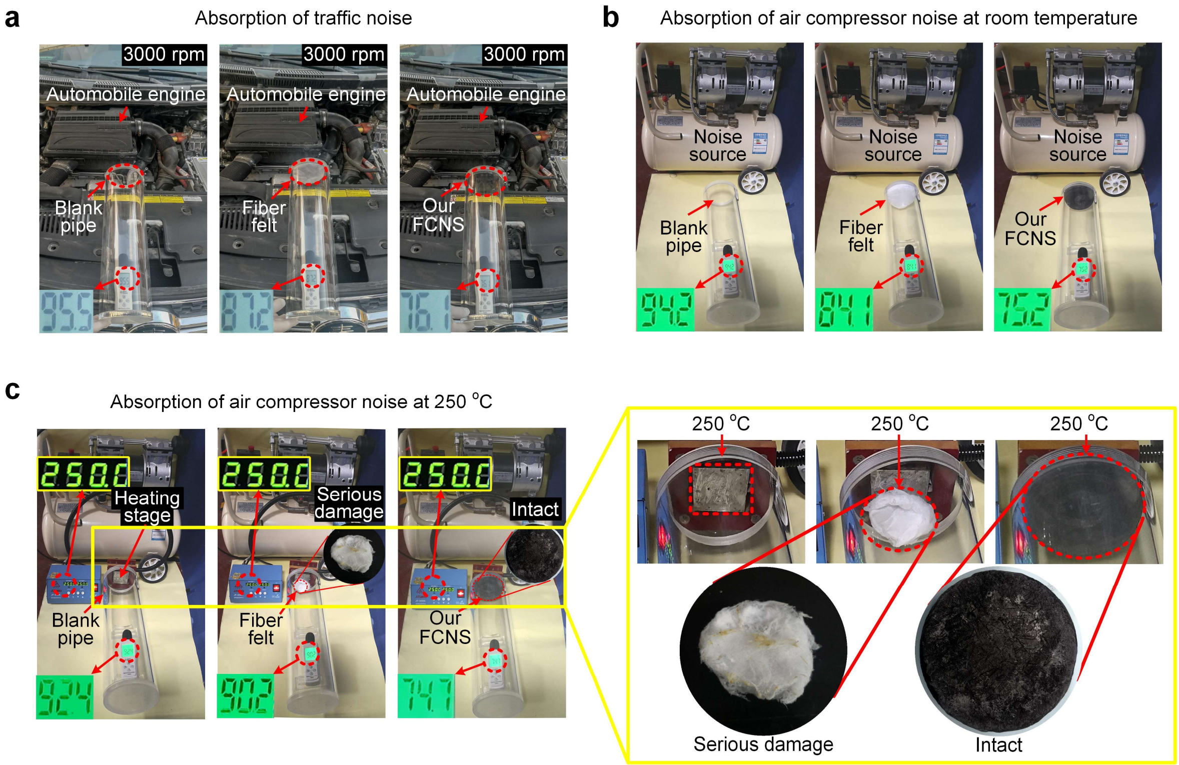

Supplementary Fig. 17 Photographs showing the direct application of the commercial fiber felt and sandwiched FCNS on the (**a**) automobile engine noise absorption at 3000 rpm, (**b**) air compressor noise absorption at room temperature and (**c**) air compressor noise absorption at 250 °C.

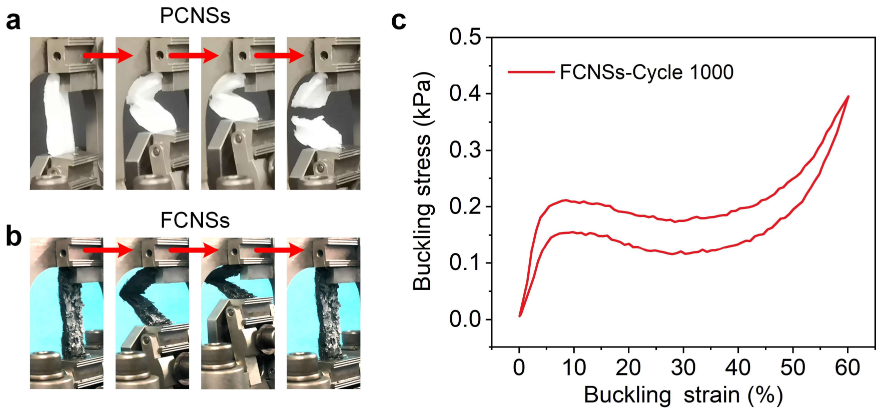

Supplementary Fig. 18 Photographs of (**a**) PCNSs and (**b**) FCNSs under a buckling-recovery cycle. (**c**) A 1000-cycle bending fatigue test of FCNSs with a buckling strain of 60%.

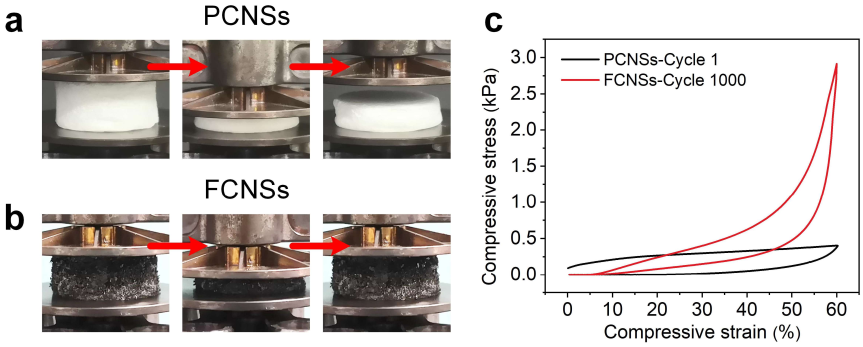

Supplementary Fig. 19 Photographs of (**a**) PCNSs and (**b**) FCNSs under a compressing and releasing cycle. (**c**) A 1-cycle fatigue test of PCNSs and 1000-cycle fatigue test of FCNSs with a compressive strain of 60%.

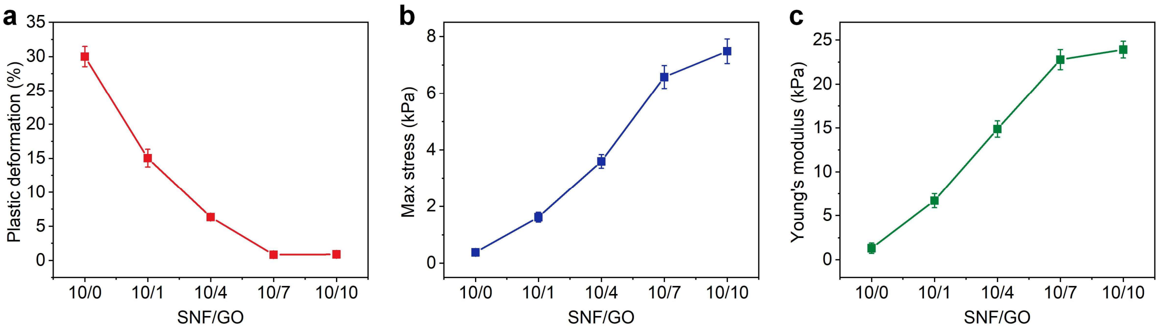

Supplementary Fig. 20 (**a**) Plastic deformation, (**b**) max stress and (**c**) Young's modulus of FCNSs with various ratios of SNF/GO. Error bars in (**a-c**) represent the standard deviations of three replicates.

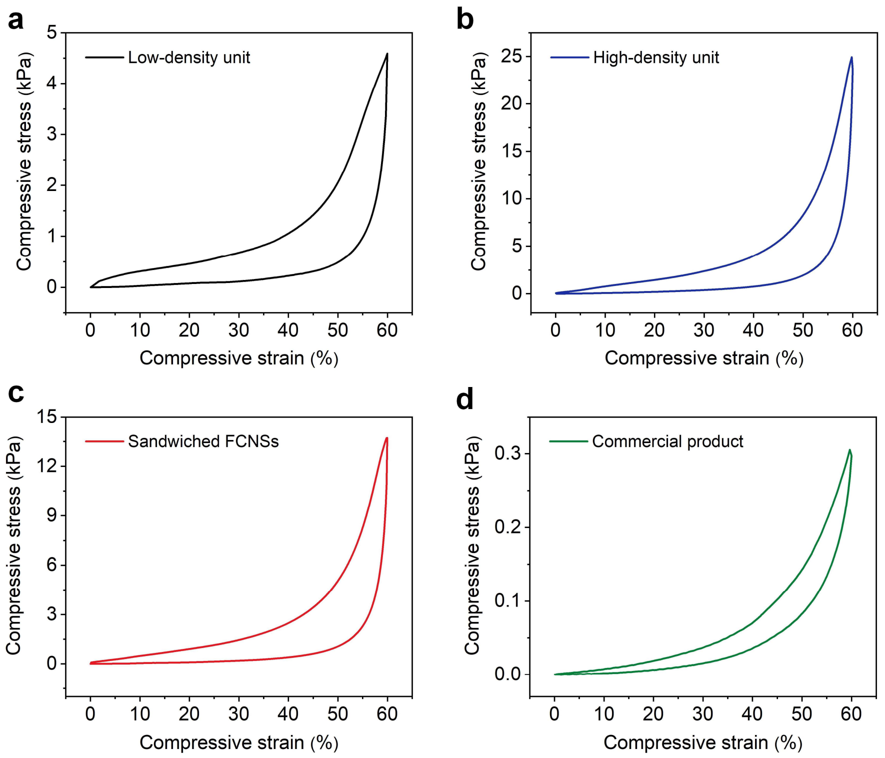

Supplementary Fig. 21 Mechanical resilience of (**a**) low-density unit, (**b**) high-density unit, (**c**) sandwiched FCNSs and (**d**) commercial product.

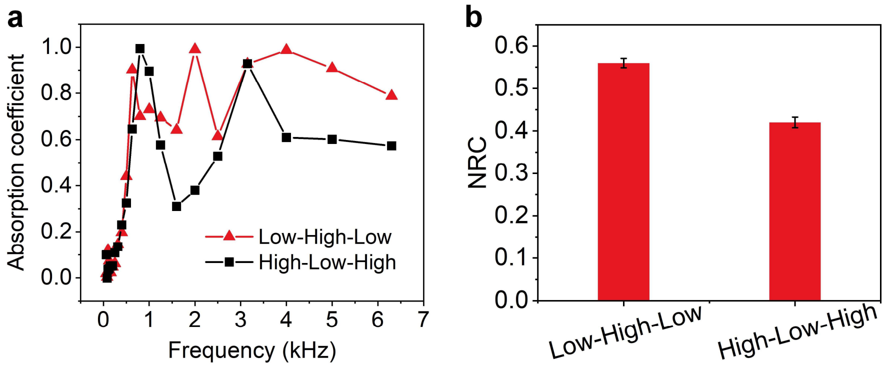

Supplementary Fig. 22 Comparison of (**a**) noise absorption coefficient and (**b**) NRC of Low-High-Low (5–18–5 mg cm^{-3}) combination and High-Low-High (18–5–18 mg cm^{-3}) combination. Error bars represent the standard deviations of three replicates.

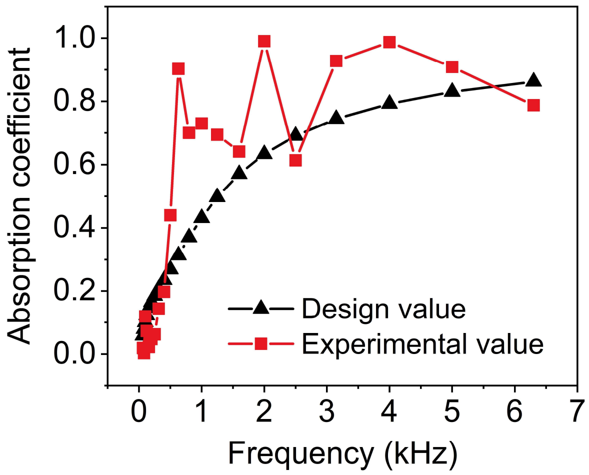

Supplementary Fig. 23 The design value and experimental value of the sound absorption coefficient of the sandwiched FCNSs.

Supplementary Table 1 Comparison of the preparation methods of noise-absorbing materials.

Preparation method	Material	Thickness (mm)	Density (mg cm^{-3})	Compressibility	NRC	Reference
Electrospun + Freeze-drying	Fibrous sponges	30	9.33	1000 cycles at 60% strain, 4.3% plastic deformation	0.56	This work
Foaming	Melamine foams	26	9.84	1 cycle at 80% strain, ~5% plastic deformation	0.35	[1]
Sol-gel	Silica aerogels	30	65–70	/	0.40	[2]
Sintering	Porous ceramic	28	670	/	0.48	[3]
Melt-drawing	Glass fibers	30	41	500 cycles at 60% strain, 10% plastic deformation	0.40	Commercial product
Melt-blowing	Fiber felts	30	17	300 cycles at 60% strain, 5.6% plastic deformation	0.48	Commercial product

Supplementary Table 2 The sound attenuation of sandwiched FCNSs and commercial products.

Materials	Thickness (mm)	Density (mg cm^{-3})	Sound attenuation (dB)
Sandwiched FCNSs	30	9.33	27
Commercial products	30	17	13.2

Supplementary Table 3 Comparison of mechanical resilience of sandwiched FCNSs, low-density units, high-density units and commercial products.

Materials	Volume density (mg cm^{-3})	Young's modulus (kPa)	Plastic deformation (%)
Sandwiched FCNSs	9.33	5.33	1.50
Low-density unit	5	4.25	0.26
High-density unit	18	7.58	1.81
Commercial product	17	0.07	2.42

Supplementary Table 4 The acoustic characteristic parameters of FCNSs with various GO amounts.

Materials	φ (%)	σ (Pa s m^{-2})	Λ (μm)	Λ' (μm)	α_∞
FCNS10	99.67	123420	44.4	27.0	1.002
FCNS40	99.49	306470	28.2	17.2	1.003
FCNS70	99.29	669750	19.1	11.6	1.004
FCNS100	99.11	545890	21.2	12.9	1.004

Supplementary Table 5 The acoustic characteristic parameters of sandwiched FCNSs.

Sandwich structures	φ (%)	σ (Pa s m^{-2})	Λ (μm)	Λ' (μm)	α_∞
Low-density unit	99.71	158350	39.2	23.8	1.001
High-density unit	98.95	994100	15.7	95.7	1.005
Low-density unit	99.71	158350	39.2	23.8	1.001

Supplementary Methods

Materials. Phosphoric acid (H_3PO_4, AR), polyvinyl acetate (PVA, 1788), tetraethyl orthosilicate (TEOS) and ascorbic acid were bought from Aladdin Co., Ltd, China. Graphene oxide (GO) powder was provided by Jiangsu XFNANO Materials Tech. Co., Ltd, China. All chemicals were directly used without any purification.

Fabrication of flexible SiO_2 nanofibers (SNFs). We prepared the SNFs by combining the sol-gel method and the electrospinning technique. Firstly, the silica sol was obtained by rapidly adding the H_3PO_4 drop by drop into the mixing solution of H_2O and TEOS in the molar ratio of 0.01:1:10, with the following stir for 8 h. Meanwhile, PVA powder was dissolved in deionized water at 80 °C and stirred for 5 h to form a PVA solution with a concentration of 10 wt%. Subsequently, the silica sol and PVA solution with the same weight were mixed and stirred for another 8 h to form a stable precursor solution. In a typical electrospinning process, the aforementioned precursor solution was transferred to the syringe with a metal tip for electrospinning, with the voltage of 17 kV, the feed rate of 1 mL h^{-1}, the spinning distance of 20 cm, the roller speed of 100 rpm, the ambient temperature of 25 ± 2 °C and the ambient humidity of 45 ± 5%, respectively. Afterward, the prepared PVA/SiO_2 hybrid nanofiber membrane was dried in a vacuum oven at 70 °C for 2 h to remove water, then it was put in a temperature-programmed muffle furnace to remove organic components and obtained SNF membranes, with the heating rate of 5 °C/min and termination temperature of 800 °C in air atmosphere.

Determination of density of FCNSs. The volume density (ρ) of the FCNSs was estimated using the followed formula:

$$\rho = {}^{m}\!/_{V} \tag{1}$$

Step-by-step design methodology of FCNSs. The appropriate underlying theory of noise absorption properties is important to predict the relationship between the structure and properties of sponges. Currently, the widely used prediction models for porous material are Delany-Bazley (DB) model and Johnson-Chamoux-Allard (JCA) model[4,5]. Since the DB model only has an independent variable of airflow resistance, it can't directly guide the regulation of the internal microstructure (density, tortuosity, etc.) of the porous material. In contrast, the JCA model is the most commonly used model for predicting

the noise absorption properties of porous materials. In this model, the noise absorption of porous material is mainly through the viscous effect and the temperature effect, which are described by equivalent density (ρ_{eq}) and equivalent bulk modulus (K_{eq}), respectively[6]. The calculation formulas are as follows:

$$\rho_{eq}(\omega) = \frac{\alpha_\infty \rho_0}{\varphi}\left(1+\frac{\sigma\varphi}{\mathrm{i}\omega\rho_0\alpha_\infty}\sqrt{1+\frac{4\mathrm{i}\alpha_\infty^2\eta\rho_0\omega}{\sigma^2\Lambda^2\varphi^2}}\right) \tag{2}$$

$$K_{eq}(\omega)=\frac{\gamma P_0}{\varphi}\left[\gamma-(\gamma-1)\left(1+\frac{8\eta}{\mathrm{i}\Lambda'^2 P_r\omega\rho_0}\sqrt{1+\frac{\mathrm{i}\rho_0\omega P_r\Lambda'^2}{16\eta}}\right)^{-1}\right]^{-1} \tag{3}$$

Based on the Eqs (2) and (3), the characteristic impedance Z_c and propagation constant k are determined as:

$$Z_c=\sqrt{\rho_{eq}K_{eq}} \tag{4}$$

$$k = \omega\sqrt{\rho_{eq}/K_{eq}} \tag{5}$$

The surface impedance Z_s is calculated as:

$$Z_s=-\mathrm{i}Z_c\cot(kt) \tag{6}$$

Finally, the normal incidence sound absorption coefficient α is calculated by:

$$\alpha=1-\left|\frac{Z_s-\rho_0c_0}{Z_s+\rho_0c_0}\right|^2 \tag{7}$$

Based on the Eqs (2-7), thickness t and five acoustic parameters are required to determine the sound absorption coefficient of porous materials, which are porosity φ, airflow resistivity σ, tortuosity factor α_∞, viscous characteristic length Λ and thermal characteristic length Λ'. For our fibrous sponge, five acoustic parameters can be calculated as follows[7-9]:

$$\begin{cases} \varphi = 1 - \dfrac{\rho}{\rho_s} \\ \sigma = \dfrac{S_1}{t}\dfrac{\Delta P}{U} \\ \alpha_\infty = {}^{1}\!\Big/\!\sqrt{\varphi} \\ \Lambda \approx \dfrac{1}{c}\left(\dfrac{8\alpha_\infty \eta}{\sigma\varphi}\right)^{1/2} \\ \Lambda' \approx \dfrac{1}{c'}\left(\dfrac{8\alpha_\infty \eta}{\sigma\varphi}\right)^{1/2} \end{cases} \tag{8}$$

as for the rectangular slit of our sponge, c = 0.78, c' = 0.5c.

(a) Density ρ. According to Eq. (8), the porosity of the material is negatively correlated with the density. Generally, the porosity of porous materials ranges from 95% to 100%. As the volume density increases, the friction probability between sound waves and sponge increases, thus improving the noise absorption property (especially in low frequency). However, when the volume density increases to a certain level, the resistance of sound energy entering the sponge increases, which increases the reflection of the sound wave, thus reducing the high-frequency noise absorption property. As the density increased from 5 to 18 mg cm^{-3}, the porosity decreased from 99.7% to 98.9%. Low-density sponges of 5 mg cm^{-3} showed good high-frequency (>1000 Hz) noise absorption while poor low-frequency (<1000 Hz) noise absorption. On the contrary, high-density sponges of 18 mg cm^{-3} exhibited good low-frequency noise absorption while poor high-frequency noise absorption. Therefore, we combined low-density and high-density sponges to achieve broadband efficient noise absorption.

(b) Thickness t. Thickness is the most direct parameter to reflect the noise absorption property of the sponge. With the increase of the thickness, the sound absorption coefficient of the sponge gradually increases, and the absorption peak of the sponge moves towards low frequency. Due to the limitation of installation space, the thickness of noise-absorbing material is usually between 10–40 mm. Inspired by the influence of density and thickness on the noise absorption of the sponge, we further constructed the sandwiched fibrous sponge with the combination of low-density and high-density units.

(c) Airflow resistivity σ. The airflow resistivity represents the resistance of the sponge to airflow per unit thickness, which is usually measured by the standard method. With the increase of flow resistance, the friction and viscous force increase when sound waves propagate inside the sponge, thus improving the noise absorption property. However, when the airflow resistivity is too high, it would be

difficult for sound waves to enter the sponge, which will reduce the noise absorption property. Generally, the optimal airflow resistivity of porous materials is between 10^3 and 10^7 Pa s m^{-2}.

(d) Tortuosity factor α_∞. The tortuosity factor of the sponge is related to the complexity of the internal skeleton structure, which is used to characterize the complexity of the sound wave propagation path in the sponge. When the porosity of the sponge is close to 100%, the tortuosity factor α_∞ is also close to 1.

(e) Viscous characteristic length Λ. The viscosity characteristic length describes the viscous and inertia effect between the internal air and the sponge skeleton. With the decrease of viscosity characteristic length, the viscosity resistance and friction effect of the sponge increases, resulting in more sound energy loss.

(f) Thermal characteristic length Λ'. The thermal characteristic length describes the heat exchangeability between the internal air and the sponge skeleton. The larger contact area between the internal air and the sponge skeleton will increase the heat exchangeability of the material, thereby improving the noise absorption property of the material.

In terms of material sound attenuation, the sound attenuation of the sponge is determined by the imaginary part of the sound wave propagation constant k, so Eq. (5) is written in the complex form[10]:

$$k = k_r + \mathrm{i}k_i \tag{9}$$

The t-dependence of the complex sound pressure amplitude is given by:

$$|p(t)|/|p(0)| = \exp(-k_i t) \tag{10}$$

where $p(0)$ and $p(\mathrm{t})$ are the sound pressure at the distance of 0 and t when the sound wave enters the sponge, respectively. Therefore, the corresponding attenuation L in dB at the distance t is:

$$L = 20\log_{10}|p(0)/p(t)| = 20\log_{10}(\mathrm{e})k_i t \approx 8.72k_i t \tag{11}$$

It can be seen from Eqs (2-5) and (9-11) that, the sound attenuation of the sponge is related to the thickness and the five acoustic characteristic parameters. With the increase of thickness, the sound attenuation of the sponge increases. Due to the limitation of installation space when the noise-absorbing material is used, the thickness is usually controlled at about 30 mm. Compared with commercial noise-absorbing materials, the sound attenuation of our sponge was doubled under the same test conditions, as shown in Supplementary Table 2. This is because our sponge had hierarchical structures of Low-High-Low combination and alternating flow resistance. When sound waves passed through the sandwiched sponge, stronger viscous and thermal effects made sound energy to be greatly absorbed and attenuated.

All in all, existing noise absorption theory based on isotropic uniform structures only can partly guide the design of our anisotropic fibrous sponge, and there is no appropriate theoretical formula that can perfectly fit with the hierarchical structure. However, the noise-absorbing materials with uniform structures already can't achieve further improvement of performance, especially for low-frequency noise. To overcome this drawback, complicated hierarchical structures must be created to achieve the hierarchical dissipation of broadband noise, thus improving noise absorption; and our results just prove this idea.

In addition, to ensure good long-term noise absorption performance, the fibrous sponge also needs to have certain mechanical properties. The relationship between Young's modulus (E) and bulk density (ρ) of porous materials is as follows[11,12]:

$$E = A\rho^n \tag{12}$$

To avoid the influence of the material itself, Eq. (12) is optimized by:

$$E/E_s = B\left(\rho/\rho_s\right)^n \tag{13}$$

When the material is determined, E_s and ρ_s are both fixed values. For most of the existing lightweight foams and aerogels, their mechanical properties and bulk density followed the relationship of $E/E_s \sim (\rho/\rho_s)^{2.5}$ (Fig. 2g). While for our fibrous sponges, Young's modulus and bulk density followed $E/E_s \sim (\rho/\rho_s)^{0.7}$, which indicated that the modulus of our sponge was less dependent on density, that is, our sponge can have better mechanical strength even at low density. This was because the lamellar closed-cell wall of the sponge can effectively transfer the stress. In addition, Young's modulus of our fibrous sponge was much greater than commercially available noise-absorbing materials, as shown in Supplementary Table 3, indicating that it had sufficient mechanical properties in practical applications.

Besides, the Poisson's ratio v of the material is the negative ratio of the transverse strain to the longitudinal strain, so it is independent of relative density and only depends on the pore structures of the material[13]. When the preparation method and structure are similar, the Poisson's ratio of the same material has little difference. Its expression is:

$$v = -\varepsilon_x/\varepsilon_y \tag{14}$$

The Poisson's ratio of the material is generally determined by combining experiment and calculation. As shown in Supplementary Fig. 7c, the Poisson's ratio of our sponge was in the range of –0.1 to 0. This unique negative Poisson's ratio characteristic was due to the equiaxial reversal of the

hierarchically entangled structure on the cell wall during compression, thus the sponge maintained the strain characteristics of negative Poisson's ratio when stressed.

In summary, based on the above basic theoretical formulas, we have adjusted the noise absorption properties and mechanical properties of the fibrous sponges by their controllable formation. Therefore, the sponges also had good mechanical properties on the premise of ensuring excellent noise absorption.

Calculation of sound pressure and acoustic impedance. The calculation of specific surface acoustic impedance and sound pressure was conducted using COMSOL software based on the JCA model. First, as for the FCNSs with various GO loading amounts, four cylinders with a basal diameter of 3 cm and a height of 1 cm were modeled, respectively; as for the sandwiched FCNSs, three cylinders with a basal diameter of 3 cm and a height of 1 cm were simultaneously modeled. Subsequently, five parameters were necessary to calculate the absolute sound pressure of the FCNSs, which included porosity (φ), flow resistance rate (σ), viscous characteristic length (Λ), thermal characteristic length (Λ') and tortuosity factor (α_∞). The five parameters calculated in advance were as shown in Supplementary Tables 4 and 5. Additionally, the calculated absolute sound pressure distributions in typical 250, 1000 and 2000 Hz of FCNS-5, FCNS-18 and sandwiched FCNSs were shown in Supplementary Fig. 15. The results were similar to that of 500 Hz in Figs 5e-g, which showed that the sound pressure distribution mainly depended on the characteristic parameters of the FCNSs.

Determination of thicknesses, number of layers and pore sizes of sandwiched FCNSs. Considering that the key parameters affecting the noise absorption of sponges are the porosity (depend on density) and thickness, we first analyzed the effects of volume densities (5–18 mg cm^{-3}) and thicknesses (10–40 cm) on the noise absorption of sponges. The results showed that low-density sponges showed good high-frequency (>1000 Hz) sound absorption while poor low-frequency (<1000 Hz) sound absorption. On the contrary, the high-density sponge exhibited good low-frequency sound absorption while poor high-frequency sound absorption. Moreover, the noise absorption performance of the sponges is enhanced with the increase in thickness.

In terms of the choice of thickness, considering that the space available for installation of noise-absorbing materials is limited in practical application, and the thickness of commercially noise-absorbing materials is usually about 30 mm. To facilitate practical application and comparison with commercial materials, we set the thickness of the sponge at 30 mm.

In terms of determining the number of layers, combining the effect of thickness with volume densities, we envisaged combining low- and high-density units to prepare sponges with both desirable high-frequency and low-frequency performance. Considering that there are many incident directions of noise waves in practical applications, we want to design a universal material so that sound waves can be absorbed whether they come from the front or the back. Therefore, we analyzed the noise absorption properties of low-density and high-density units in different combinations (5–18–5 mg cm^{-3} and 18–5–18 mg cm^{-3}), as shown in Supplementary Fig. 22. Compere with the combination of "High-Low-High" (NRC of 0.42), the combination of "Low-High-Low" possessed both good low-frequency and high-frequency noise absorption performance simultaneously (NRC of 0.56). Moreover, compared with the "Low-High-Low" combination (density of 9.33 mg cm^{-3}), the density of the "High-Low-High" combination increased by 1.5 times (13.67 mg cm^{-3}), which would greatly increase fuel consumption, violating the principle of energy saving. Further increasing the number of layers to 5 layers would increase the difficulty and cost of preparation, so we set the number of unit layers as 3 layers, that is, the thickness of each unit was 10 mm.

In terms of the determination of pore size, as for our sponges with lamellar structures, the pore size of the sponge was inversely proportional to the volume density. Therefore, the pore size of sponges was further determined by regulating density. When the volume density of the sponge increased from 5 to 18 mg cm^{-3}, the pore size of the sponge decreased from 100 to 10 μm.

Based on the above-mentioned theory and the flexibility of the preparation method, all the thickness, the number of layers and pore size can be easily adjusted.

Supplementary Discussions

Formation mechanism of hierarchically entangled structures. The phase transition process and forming mechanism of the hierarchically entangled structures of the FCNSs during the freeze molding process were shown in Supplementary Fig. 6. First, the phase transition of solvent during the freeze-drying process was presented in Supplementary Fig. 6a. The GO nanosheets and SiO_2 nanofibers were uniformly dispersed in water after high-speed stirring, and the water solidified into ice and formed ice crystals during the directional freezing process of liquid nitrogen. As the growth of ice crystals, the graphene nanosheets and ceramic nanofibers in the dispersion gathered along the tip of the ice crystals and were squeezed between the two icicles. Three kinds of microstructures were formed between two adjacent fibers when the well-dispersed short fibers were squeezed out by ice crystals, which involved

parallel, cross and approximate T-shape. Meanwhile, the graphene oxide nanosheets in the dispersion were gradually assembled around fibers under the extrusion of ice crystal and strong π-π interaction, which uniformly entangled on the surface of the ceramic nanofiber and firmly connected adjacent fibers. Therefore, three physical entangled structures including bridging, soldering and jointing were achieved. After freeze-drying, the ice crystals directly sublimated, and the GO nanosheets and SiO_2 nanofibers formed the hierarchically entangled structures.

Formation mechanism of closed-cell walls. The closed-cell walls of FCNSs were achieved by combining the directional freezing method and the assembly characteristics of the materials. The graphene nanosheets and ceramic nanofibers were continuously squeezed by the bottom-up ice crystals during the directional freezing process. When the content of the two materials in the dispersion reached a critical value (the mass ratio of SNF/GO was 10/7), the extrusion of ice crystals and the π-π interaction between the graphene made it completely assembled on the surface of the nanofibers and firmly connected adjacent nanofibers. Therefore, the closed-cell walls were formed along with the sublimation of ice crystals.

Preparation methods of noise-absorbing materials. The preparation methods of noise-absorbing materials mainly included freeze-drying, foaming, sol-gel, sintering, melt-drawing and melt-blowing. Our preparation method belongs to the freeze-drying method. The difference between our method and others was shown in Supplementary Table 1. Compared with uniform structures of noise-absorbing materials prepared by other methods, the unique hierarchically entangled structures endowed our sponges with a large number of effective bonding points; meanwhile, it also provided hierarchical reflections for sound waves inside the sponges and caused more energy dissipation. Therefore, our FCNSs possessed both better mechanical properties and noise absorption properties simultaneously.

Structural tunability of FCNSs and freeze-drying method. To fabricate sponges with desirable mechanical and noise absorption properties, we directly regulated the density, thickness and mass ratio of ceramic nanofibers and graphene. On this basis, we indirectly adjusted pore size, Young's modulus and Poisson's ratio of sponges. The density of sponges was regulated from 2 to 20 mg cm^{-3}, the thickness of sponges was regulated from 10 to 40 mm, the pore size of sponges was regulated from 5 to 100 μm, the mass ratios of ceramic nanofibers and graphene were regulated from 10/1 to 10/10, Young's

modulus was regulated from 1 to 25 kPa and the Poisson's ratio of the sponges was always maintained at –0.1 to 0.

Freeze drying is to freeze the liquid in the internal pores of the wet gel network, and then the frozen crystals are directly sublimed in a vacuum state. There is no gas-liquid interface during the whole process, and thus no capillary force to cause the structure collapse. Therefore, the original structure and morphology were maintained when the sponge was achieved. The freeze-drying method can achieve certain structural parameters by controlling the dispersion parameters (solid content and weight) and freezing conditions (freezing rate, temperature and freezing time). In terms of dispersion parameters, the volume density of the sponge can be precisely controlled by regulating the solid content (graphene and ceramic nanofibers) of the dispersion, and the thickness of the sponge can be precisely controlled by regulating the weight of the dispersion. Moreover, the pore size of the sponge is inversely proportional to the solid content of the dispersion. This is because the higher solid content in the dispersion increases the resistance of the growth of ice crystals, resulting in smaller crystal size, and thus the sponge has a smaller pore size. As for freezing conditions, the pore size of the sponge is inversely proportional to the freezing rate, which is because a faster freezing rate accelerates the growth of crystal nuclei, and the nucleation rate is greater than the crystal growth rate. This leads to the smaller size of the crystal, thus reducing the pore size of the sponge. Moreover, lower freezing temperature and faster freezing rate are conducive to the formation of a lamellar structure, which is because the faster growth rate of ice crystal makes the columnar ice crystals fuse into a lamellar structure. Additionally, a longer freezing time will also gradually fuse the columnar ice crystals into layers.

Mechanical resilience of FCNSs with different SNF/GO ratios. Pure ceramic nanofibrous sponges (PCNSs) were constructed by the simple accumulation of short SNF, and there was no effective bonding point between the fibers. It was easy to slip between the fibers when subjected to external forces, resulting in irreversible damage to the sponge. Therefore, PCNSs was almost no buckling property (breaking in the first bending, Supplementary Fig. 18a), and the plastic deformation of which reached 30% at the first compression cycle (Supplementary Figs 19a and c). In contrast, as for FCNSs, the graphene networks were not only cleverly entangled on the single SNF but also effectively bonded adjacent fibers together, which was equivalent to forming a large number of effective bonding points between the fibers. Moreover, the strong π-π interaction between the graphene networks further strengthened these bonding points, thereby increasing the stability of the bonding points. What's more, compared with chemical

bonding points, these physical entangled structures were stronger and more stable. Therefore, our FCNSs possessed desirable buckling (Supplementary Figs 18b-c) and compression properties (Supplementary Figs 19b-c).

As the mass ratio of SNF/GO increased from 10/0 (pure SNF sponge) to 10/10, the compressive plastic deformation of the sponges decreased from 30% to 0.8% (Supplementary Fig. 20a), indicating that the elasticity of the sponge gradually improved. Moreover, the maximum compressive stress of the sponges increased from 0.4 to 7.5 kPa (Supplementary Fig. 20b), and Young's modulus increased from 1.3 to 23.9 kPa (Supplementary Fig. 20c). Furthermore, it can be seen that the mechanical properties of mass ratios of 10/7 and 10/10 were similar. Considering that the formulation of 10/10 led to the agglomeration of graphene due to the high graphene content (Fig. 4d), which caused the nonuniform structure of sponges, and thus resulted in the decrease of noise absorption property. After comprehensive consideration, the formulation of 10/7 was chosen for subsequent experiments.

Mechanical resilience of the sandwiched FCNSs. Considering that the sandwich structures may affect the mechanical resilience of FCNSs, we tested the mechanical resilience of the low-density unit, the high-density unit and the sandwiched FCNSs, respectively. As shown in Supplementary Fig. 21, although the max stresses of several sponges varied greatly, the compressive plastic deformations of all the sponges were very small (0.26–1.81%). Moreover, all of the deformations were smaller than that of the commercial products (2.42%) under the same test conditions (Supplementary Fig. 21d), indicating that the mechanical resilience of the sponge was little affected by the sandwich structure. This is because our sponges were composed of the stable entangled structures formed by GO and SNFs, and the mechanical resilience of the sponges was not strongly dependent on the volume density.

Surprisingly, there was little difference between Young's modulus of the sandwiched and the high- and low-density unit sponges, which corresponded to the weak dependence of Young's modulus on the density, as shown in Supplementary Table 3 and Fig. 2g. For most of the existing lightweight foams and aerogels, the relationship between their mechanical properties and volume density followed $E/E_s \sim (\rho/\rho_s)^{2.5}$. While Young's modulus and volume density for our sponges followed $E/E_s \sim (\rho/\rho_s)^{0.7}$, indicating that our sponge can also have good mechanical strength at low density, which was because the closed-cell walls of the sponges can effectively transfer the stress. In addition, Young's modulus of our sponges was much larger than that of commercial noise absorption materials (Supplementary Table 3), indicating that our sponges had sufficient mechanical properties in the practical applications.

Relationship between volume density and areal density. To avoid the influence of thickness on sound absorption performance, the areal density was used to compare the NRC of noise reduction materials. By contrast, the commonly used volume density of 3D porous materials was selected to describe FCNSs in other parts. The relationship of the areal density (ρ_a, g m^{-2}) and volume density (ρ, mg cm^{-3}) is:

$$\rho_a = \rho * t \quad (15)$$

Comparison of the experimental and design values of sandwiched FCNSs. The comparison of the experimental value with the design value is very important for validating the design. Based on the above formula derived from the JCA model, to calculate the sound absorption coefficient of the sponge, we first obtained the acoustic characteristic parameters of the sandwich structure, as shown in Supplementary Table 5. Based on the calculation of the above parameters, the sound absorption coefficient curve can be obtained. The comparison of the design value and experimental value was shown in Supplementary Fig. 23, it can be seen that the law of the design value and experimental value of sound absorption coefficient of sandwiched FCNSs was similar. However, the experimental values of most frequency points were higher than the design values, and the NRC of the design value and the experimental value were 0.38 and 0.56, respectively. The reason was that the premise of using the JCA model to calculate the sound absorption coefficient on the assumption that the material is the isotropic uniform structure[14]. It was equivalent to idealizing our sandwiched sponge as a periodical uniform object in the calculation, that is, assuming that each unit was a uniform structure. Thus, the sound wave was subjected to the same flow resistance and uniform dissipation when propagated to any part of the sponge. In fact, each unit of our sandwiched sponge was an anisotropic block with a hierarchical structure, which was designed to make more sound waves enter the sponge and didn't transmit out in the propagation process. Compared with the uniform structure, the hierarchal structure not only increased the transmission path of the sound wave but also made the sound wave reflect repeatedly, thereby enhancing the sound absorption coefficient. Moreover, when sound waves passed through the interface between low- and high-density units, due to the huge gap in flow resistance, more sound waves were reflected inside the sandwiched sponges, thus further increasing sound energy loss.

Effect of hierarchically entangled structure on sound absorption. Based on the Eqs (2-8), thickness *t* and five acoustic parameters were required to determine the sound absorption coefficient of porous

materials, which was porosity φ, tortuosity factor α_∞, airflow resistivity σ, viscous characteristic length Λ and thermal characteristic length Λ'[15]. Compared with the uniform structure, in the same thickness (10 mm), the hierarchically entangled structure significantly improved airflow resistivity (from 1.58×10^6 to 6.70×10^5 Pa s m^{-2}) and decreased porosity (from 99.8% to 99.3%) of the sponge. Thus, the noise absorption properties of the sponges were enhanced (from 0.07 to 0.27). On this basis, further constructing sandwich structure combined the advantages of density and thickness, greatly improving the noise absorption property (NRC from 0.40 to 0.56).

Dissipation of sound energy on the cavity walls. Three forms of changes happened when acoustic energy dissipated on the cavity walls[16-18]: 1) Attenuation caused by air viscosity. When sound waves propagated into the sponge, the air molecules inside the sponge vibrated continuously and rubbed with the cavity walls, and the resultant viscous resistance converted the acoustic energy into heat energy and then attenuated. 2) Dissipation by heat conduction. When sound waves passed through the porous sponge, the air particles in the pores were periodically compressed and released, so there was a temperature gradient between the air particles, which consumed part of the acoustic energy by heat conduction. 3) Dissipation caused by vibration. The vibration of the sponge cavity walls also dissipated a small part of the acoustic energy.

Supplementary References

1. Nine, M. J. *et al.* Graphene oxide-based lamella network for enhanced sound absorption. *Adv. Funct. Mater.* **27**, 1703820 (2017).
2. Buratti, C., Merli, F. & Moretti, E. Aerogel-based materials for building applications: Influence of granule size on thermal and acoustic performance. *Energ. Buildings* **152**, 472-482 (2017).
3. Duan, C. Y., Cui, G., Xu, X. B. & Liu, P. S. Sound absorption characteristics of a high-temperature sintering porous ceramic material. *Appl. Acoust.* **73**, 865-871 (2012).
4. Kino, N. & Ueno, T. Improvements to the Johnson-Allard model for rigid-framed fibrous materials. *Appl. Acoust.* **68**, 1468-1484 (2007).
5. Allard, J. F. & Champoux, Y. New empirical equations for sound propagation in rigid frame fibrous materials. *J. Acoust. Soc. Am.* **91**, 3346-3353 (1992).
6. Allard, J. F. *Propagation of sound in porous media: Modelling sound absorbing materials* (John

Wiley & Sons, 2009).

7. Kino, N. & Ueno, T. Experimental determination of the micro- and macrostructural parameters influencing the acoustical performance of fibrous media. *Appl. Acoust.* **68**, 1439-1458 (2007).
8. Kino, N. & Ueno, T. Comparisons between characteristic lengths and fibre equivalent diameters in glass fibre and melamine foam materials of similar flow resistivity. *Appl. Acoust.* **69**, 325-331 (2008).
9. Ayub, M., Nor, M. J. M., Fouladi, M. H., Zulkifli, R. & Amin, N. A practical acoustical absorption analysis of coir fiber based on rigid frame modeling. *Acoust. Phys+.* **58**, 246-255 (2012).
10. Ingard, U. *Noise reduction analysis* (Jones & Bartlett Publishers, 2009).
11. Wu, Y. *et al.* Three-dimensionally bonded spongy graphene material with super compressive elasticity and near-zero Poisson's ratio. *Nat. Commun.* **6**, 6141 (2015).
12. Zhang, Q. *et al.* Hyperbolically patterned 3D graphene metamaterial with negative Poisson's ratio and superelasticity. *Adv. Mater.* **28**, 2229-2237 (2016).
13. Gibson, L. J. & Ashby, M. F. *Cellular solids: Structure and properties* (Cambridge Univ. Press, 1997).
14. Mosanenzadeh, S. G., Doutres, O., Naguib, H. E., Park, C. B. & Atalla, N. A numerical scheme for investigating the effect of bimodal structure on acoustic behavior of polylactide foams. *Appl. Acoust.* **88**, 75-83 (2015).
15. Cao, L. *et al.* Ultralight, superelastic and bendable lashing-structured nanofibrous aerogels for effective sound absorption. *Nanoscale* **11**, 2289-2298 (2019).
16. Cao, L. T., Fu, Q. X., Si, Y., Ding, B. & Yu, J. Y. Porous materials for sound absorption. *Compos. Commun.* **10**, 25-35 (2018).
17. Tang, X. N. & Yan, X. Acoustic energy absorption properties of fibrous materials: A review. *Compos. Part A* **101**, 360-380 (2017).
18. Peng, L., Song, B., Wang, J. & Wang, D. Mechanic and acoustic properties of the sound-absorbing material made from natural fiber and polyester. *Adv. Mater. Sci. Eng.* **2015**, 274913 (2015).
