# Supplementary material for: Flexible ceramic nanofibrous sponges with hierarchically entangled graphene networks enable noise absorption
Source: Nat Commun. 2021 Nov 15;12:6599. doi: 10.1038/s41467-021-26890-9 (PMC8593031; doi:10.1038/s41467-021-26890-9)
Supplement: Supplementary file 2 — Description of Additional Supplementary Files [file 41467_2021_26890_MOESM2_ESM.pdf]

Description of Additional Supplementary Files

File Name: Supplementary Movie 1
Description: Buckling and recovery processes of FCNSs

File Name: Supplementary Movie 2
Description: Compression and recovery processes of FCNSs.

File Name: Supplementary Movie 3
Description: Compression testing in liquid nitrogen.
